# Coordination Modes and Binding Patterns in Lanthanum Phosphoramide Complexes

**DOI:** 10.1021/acs.inorgchem.3c04521

**Published:** 2024-03-06

**Authors:** Andrew C. Boggiano, Maximilian G. Bernbeck, Ningxin Jiang, Henry S. La Pierre

**Affiliations:** † School of Chemistry and Biochemistry, Georgia Institute of Technology, Atlanta, Georgia 30332-0400, United States; ‡ Nuclear and Radiological Engineering and Medical Physics Program, School of Mechanical Engineering, Georgia Institute of Technology, Atlanta, Georgia 30332-0400, United States

## Abstract

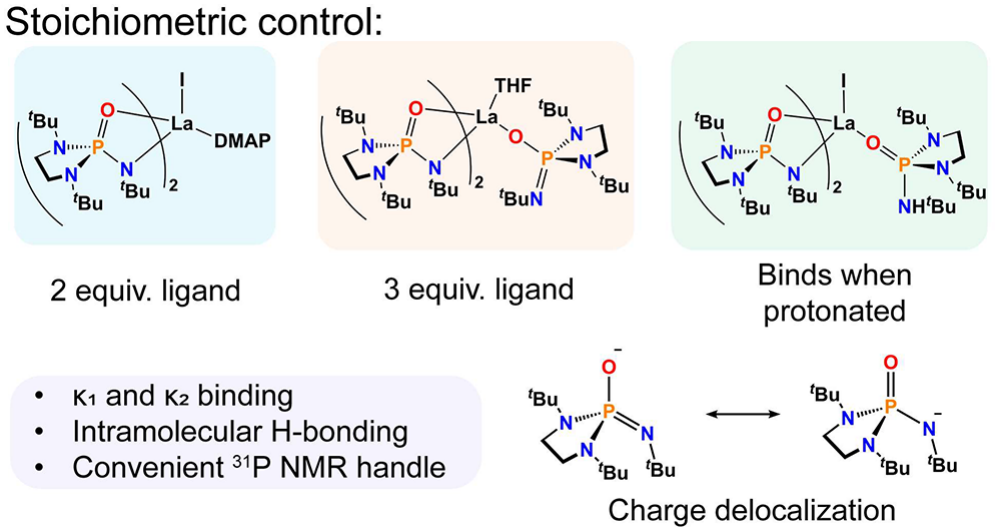

A monoanionic phosphoramide ligand is introduced, which forms a series of lanthanum complexes with the ligand in both anionic and neutral forms. Stoichiometric control alone provides monometallic complexes with either two or three phosphoramide ligands. Alternatively, a combination of anionic and neutral proteo ligands featuring intramolecular hydrogen bonding can be obtained. The anionic form of the ligand binds lanthanum as a bi- or monodentate ligand, depending on the steric demand at the metal center, while the protonated ligand binds exclusively through the phosphoramide oxygen donor.

## Abstract 2

A phosphoramide ligand is described, which binds to La^3+^ in its protonated and deprotonated forms. Stoichiometric control alone provides a variety of heteroleptic, monometallic complexes of La^3+^.

## Introduction

Due to largely ionic binding in the lanthanides compared to transition or actinide metals,^1^ many ligands ubiquitous in transition metal chemistry, such as phosphines and N-heterocyclic carbenes (NHCs), have a much lower binding affinity for the lanthanides.^2,3^ According to hard–soft acid base theory,^4^ lanthanides form hard cations while ligands such as phosphines are considered soft, making the pairing much less favorable when compared to the pairing with the transition metals (although there are exceptions to this general observation^2,5−14^). Ligands incorporating phosphorus are advantageous for the study of paramagnetic lanthanide complexes, due to the 100% abundant spin ^1^/_2_ nucleus, ^31^P. This spectroscopic feature provides a ready handle for nuclear magnetic resonance (NMR) spectroscopy for the facile screening of reaction outcomes and solution characterization of products.

The La^3+^ ion is of interest for catalytic applications, owing to its Lewis acidity and low toxicity compared to those of conventional metal catalysts.^15−18^ The ionic character of metal–ligand bonds results in reactive moieties (an attractive property for catalysis); however, the large ionic radius of La^3+^ (1.03 Å)^19^ requires bulky and/or chelating ligands to prevent deleterious reactions such as ligand redistribution or disproportionation that may proceed through complex dimerization.^20,21^ The inclusion of intramolecular hydrogen bonding interactions may serve to inhibit these processes. Herein, an anionic ligand that demonstrates rich heteroleptic, hemilabile coordination chemistry at the largest of the rare earth ions is presented. The complexes were found to be tolerant to protonation while maintaining mononuclear speciation. Intramolecular hydrogen bonding may be an important element discouraging disproportionation and/or dimerization, and similar hydrogen bonding interactions have been shown to template the synthesis of otherwise inaccessible copper–rare earth multimetallic complexes.^22^ The possibility of using hydrogen bonding in the secondary coordination sphere to support reactive moieties at large, early lanthanide metal centers is considered, as has been applied in first-row transition metal complexes^23,24^ and a Ce^4+^–oxo complex.^25^

We previously described^26,27^ π donation from the dialkyl amido substituents at phosphorus as key interactions contributing to the high basicity and donor ability of imidophosphorane ligands. Here, a related phosphoramide is reported, which incorporates *tert*-butyl amide at phosphorus, allowing for a site to deprotonate to afford a monoanionic ligand. To explore how the ligand binds to lanthanides, the closed-shell La^3+^ ion was chosen. Stoichiometric control alone allows for the isolation of a range of complexes featuring either two or three ligands, with the latter including complexes featuring a combination of neutral (protonated) and anionic (deprotonated) ligands in the primary coordination sphere.

## Results and Discussion

### Synthesis

**Figure 1:**
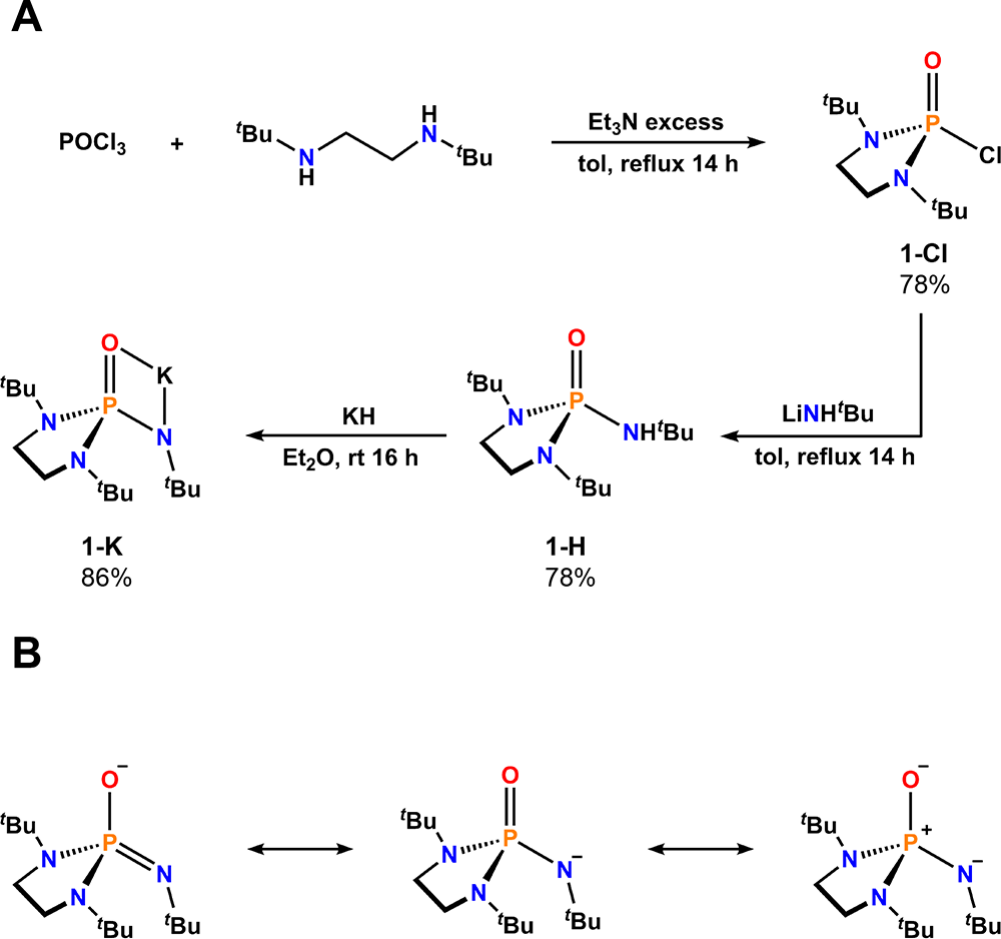
(A) Synthetic scheme. (B) Resonance representations of **1**^**–**^.

The proteo ligand, O=P(*N*,*N*′-di-^*t*^Bu-ethylenediamide)(NH^*t*^Bu) (**1-H**, ^*t*^Bu = *tert*-butyl), was prepared in two steps from commercially available starting materials (Figure 1A). The synthesis of O=P(*N*,*N*′-di-^*t*^Bu-ethylenediamide)Cl (**1-Cl**) was unexpectedly difficult but ultimately achieved by heating POCl_3_ and *N*,*N*′-di-*tert*-butylethylenediamine in toluene at reflux. After the volatiles were removed, the crude solid was purified via vacuum sublimation to give **1-Cl** in 78% yield at a 30 g scale (see Experimental Details). The primary amide substituent was installed by reaction of **1-Cl** with ^*t*^BuNHLi, with heating in toluene. The P–Cl bond in **1-Cl** was found to be rather unreactive; no reaction was observed between reflux of ^*t*^BuNH_2_ and **1-Cl** in toluene, and even starting with ^*t*^BuNHLi, we found the reaction did not proceed at room temperature. The relative inertness of the P–Cl bond in **1-Cl** was exemplified by its stability under ambient conditions, and the lack of reactivity observed upon dissolution of **1-Cl** in acetone or methanol. **1-H** was deprotonated by potassium hydride or benzyl potassium (KBn) and could be isolated as either the THF (tetrahydrofuran) solvate [K(THF)_*x*_][O=P(*N*,*N*′-di-*tert*-butylethylenediamide)(N^*t*^Bu)] [**1-K**_**THF**_; *x* ∼ 0.5–1.5 (see Experimental Details)] or the solvent-free salt [K][O=P(*N*,*N*′-di-*tert*-butylethylenediamide)(N^*t*^Bu)] (**1-K**). Both **1-K**_**THF**_ and **1-K** were determined to be competent starting materials for further reactions.

**Figure 2:**
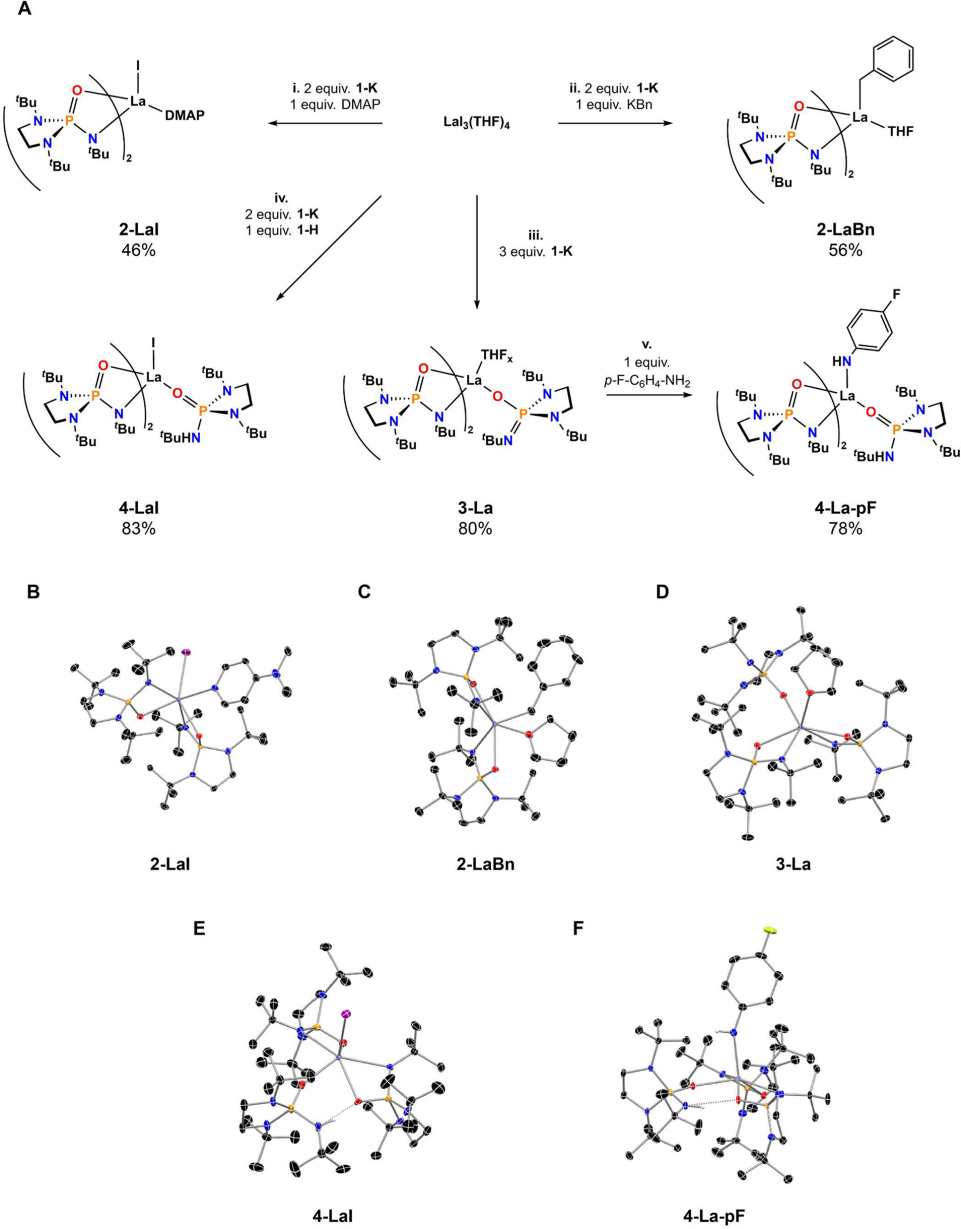
(A) Synthetic overview, under the following conditions: (i) diethyl ether, 16 h, −2KI; (ii) toluene, 16 h, −3KI; (iii) diethyl ether, 16 h, −3KI; (iv) diethyl ether, 16 h, −3KI; (v) THF, 1 h, −196 °C to rt. Structures derived from SC-XRD data of (B) **2-LaI**, (C) **2-LaBn**, (D) **3-La**, (E) **4-LaI**, and (F) **4-La-pF**. Thermal ellipsoids displayed at 50% probability. Hydrogen atoms bound to carbon and C atom disorder are omitted for the sake of clarity. Legend: lilac for La, purple for I, orange for P, yellow for F, red for O, blue for N, black for C, and white for H.

Metathesis between 2 equivalents of **1-K** and LaI_3_(THF)_4_ and 1 equivalent of *N,N*-dimethyl-4-aminopyridine (DMAP) in diethyl ether overnight produced the iodide complex, La(O=P(*N*,*N*′-di-*tert*-butylethylenediamide)(N^*t*^Bu))_2_(DMAP)I (**2-LaI**) [(Figure 2A(i)]. The metathesis reaction between 2 equivalents of **1-K** and LaI_3_(THF)_4_ in diethyl ether with the addition of 1 equivalent of benzyl potassium yielded the organometallic complex, La(O=P(*N*,*N*′-di-*tert*-butylethylenediamide)(N^*t*^Bu))_2_(THF)(CH_2_C_6_H_5_) (**2-LaBn**), incorporating 2 equivalents of **1**^**–**^, as the THF adduct [Figure 2A(ii)]. The THF adduct of the tris-ligated complex, La(O=P(*N*,*N*′-di-*tert*-butylethylenediamide)(N^*t*^Bu))_3_(THF)_*x*_ (**3-La**), was obtained by metathesis between 3 equivalents of **1-K** and LaI_3_(THF)_4_ in diethyl ether [Figure 2A(iii)]. A reaction analogous to the synthesis of **3-La**, replacing 1 equivalent of **1-K** with **1-H**, yielded the monoiodide complex with two anionic ligands and one protonated ligand, La(O=P(*N*,*N*′-di-*tert*-butylethylenediamide)(N^*t*^Bu))_2_(O=P(*N*,*N*′-di-*tert*-butylethylenediamide)(NH^*t*^Bu))I·(C_7_H_8_)_0.5_ (**4-LaI**) [Figure 2A(iv)]. A mixed-protonation-state complex could also be synthesized via the acid–base reaction of **3-La** with *p*-fluoroaniline in THF, yielding the complex La(O=P(*N*,*N*′-di-*tert*-butylethylenediamide)(N^*t*^Bu))_2_(O=P(*N*,*N*′-di-*tert*-butylethylenediamide)(NH^*t*^Bu))((4–F-C_6_H_4_)NH)·THF (**4-La-pF**) [Figure 2A(vi)]. The basicity of the ligand limited the scope of this acid–base reaction, as an analogous reaction with ^*t*^BuNH_2_ yielded only **3-La** after the volatiles had been removed. Overall, a family of heteroleptic complexes could be accessed through one-step syntheses.

The hydrogen bonding interactions observed in **4-LaI** and **4-La-pF** suggested the possibility of stabilizing reactive metal-bound fragments, such as -oxo or -imido moieties, via hydrogen bonding with the secondary coordination sphere of the complexes. This approach has been demonstrated in the transition metal literature.^23,24,28,29^ In the lanthanides, this strategy led to stabilization of a rare terminal Ce^4+^–oxo complex.^25^ The closed-shell, [Xe] electronic configuration of La^3+^ precluded the synthesis of a metal–imido complex by metal-centered two-electron azide reduction. However, the deprotonation of a metal–anilido complex with no formal change in the metal oxidation state is an alternative route and has been demonstrated in the generation of Ce^4+^–imido complexes.^30,31^ The terminal imido functional group is rare in lanthanide complexes,^30−34^ with no examples at lanthanum. **4-La-pF** was evaluated as a precursor for generating a La^3+^–imido complex via deprotonation using benzyl potassium. **4-La-pF** contains two labile protons: one at the anilide substituent and another that is engaged in hydrogen bonding across two ligands.

**Scheme 1:**
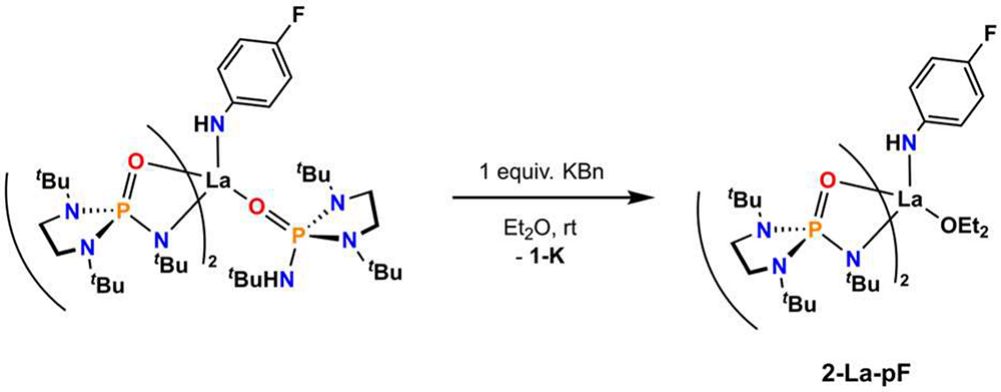
Attempted Deprotonation of **4-La-pF** (see Experimental Details for further details)

**Figure 3:**
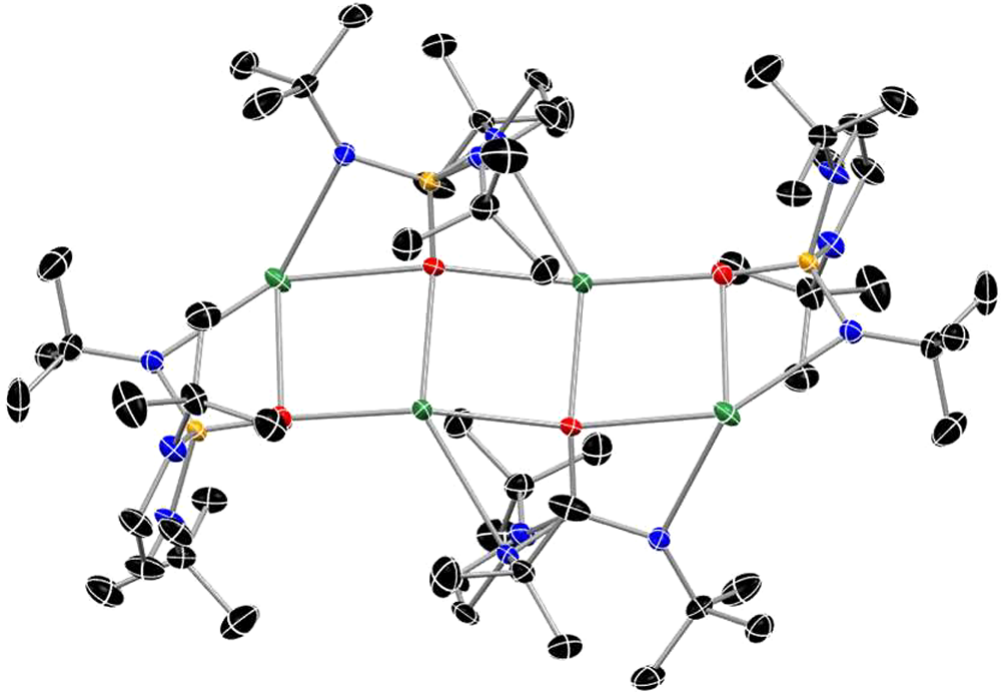
Structure of **1-K** derived from SC-XRD data. The asymmetric unit is half of the tetrameric structure shown. Legend: green for K, orange for P, red for O, blue for N, and black for C. Thermal ellipsoids displayed at 50% probability. Hydrogen atoms and C atom disorder are omitted for the sake of clarity.

Reaction of **4-La-pF** with 1 equivalent of benzyl potassium resulted in the elimination of 1 equivalent of **1-K**, producing the complex La(O=P(*N*,*N*′-di-*tert*-butylethylenediamide)(N^*t*^Bu))_2_(*p*-F-C_6_H_4_-NH) (**2-La-pF**), which was structurally authenticated via single-crystal X-ray diffraction (SC-XRD), instead of a deprotonated form of **4-La-pF** (Scheme 1). The mechanism of formation was not evident; however, evidence for the formation of a transient terminal lanthanum phosphinidene was reported via a similar acid–base approach.^35^ The thermodynamic stability of the tetrameric structure of **1-K** in the solid state (Figure 3) was likely the driving force preventing the formation of anionic, sterically congested complexes and favoring the elimination **1-K**. This hypothesis was supported by the reaction of **3-La** with 1 equivalent of benzyl potassium in diethyl ether, which resulted in the production of **1-K** and **2-LaBn** (see Experimental Details for further details).

### Structural Analysis

The P–N bonds can be classified by the number of alkyl substituents on nitrogen; herein, N^2°^ is used to refer to the di-^*t*^Bu-ethylenediamide backbone nitrogen atoms, and N^1°^ is used to denote the nitrogen of the -NH^*t*^Bu substituent that participates in metal coordination. The P–N and P–O bond lengths in **1-Cl**, **1-H**, and **1-K** display trends that represent the variable electronic localization and donation within the ligand framework. Generally, P–N^2°^ bond lengths for the backbone are longer than P–N^1°^ distances, consistent with the steric constraints at the N^2°^ site. The average P–O bond length is shortest [1.465(2) Å] in **1-Cl** and longest in **1-K** [1.520(3) Å], with **1-H** displaying an intermediate P–O distance [1.490(1) Å (Table 1)].

**Table 1:** Averaged Structural Metrics and Parameters Derived from SC-XRD Data^a^

	**1-Cl**	**1-H**^b^	**1-K**	**2-LaI**^c^	**2-LaBn**^c^	**2-La-pF**	**3-La**^c^	**4-LaI**^d^	**4-La-pF**^d^
P–O (Å)	1.465(2)	1.490(1)	1.520(3)	1.538(4)	1.530(2)	1.528(1)	1.526(1)^f^	1.533(4)	1.531(3)
							1.549(1)^g^		
P–N^1°^ (Å)	–	1.638(2)	1.568(9)	1.595(4)	1.595(4)	1.602(5)	1.560(1)^f^	1.626(5)	1.592(7)
							1.606(2)^g^		
P–N^2°^ (Å)^e^	1.634(4)	1.674(2)	1.71(2)	1.674(5)	1.678(10)	1.680(12)	1.682(12)^f^	1.656(11)	1.678(9)
							1.707(4)^g^		
P–N^2°†^ (Å)	1.634(4)	1.675(1)	1.693(2)	–	–	1.670(5)	–	1.674(10)	1.671(2)
P–N^2°Δ^ (Å)	–	1.672(1)	1.72(2)	–	–	1.689(6)	–	1.685(7)	1.689(2)
∑N°^†^ (deg)	355(1)	355.1(2)	359.4(4)	358(1)	359(1)	359.9(1)	359(1)	360(1)	359(1)
∑N°^Δ^ (deg)	–	350.1(2)	351(1)	350(1)	351.4(4)	351.1(3)	351(4)	354(1)	350(1)
∠O–P–N^1°^ (deg)	–	106.54(6)	109.0(9)	102.27(5)	102.05(5)	102.3(3)	102.22(2)^f^	101.99(16)	103.06(8)
							107.27(8)^g^		

a Estimated standard deviation in parentheses.

b Only one crystallographically distinct unit of **1-H** is present, and values given are individual metrics, not averages, except for P–N^2°^.

c The positional disorder of the backbone carbon atoms precludes distinction between P–N^2°Δ^ and P–N^2°†^ distances.

d The parameters and metrics for the protonated ligand equivalent are omitted.

e An average of all P–N^2°^ distances regardless of pyramidal/planar distinction, where applicable.

f Values for ligands bound in κ^1^ mode.

g Values for ligands bound in κ^2^ mode.

The deprotonation of **1-H** provides anionic **1**^**–**^ that can be considered by multiple resonance structures (Figure 1B). Where the double bond to P (i.e., P=O vs P=N^1°*t*^Bu) is depicted is largely a stylistic preference, as the nature of hypervalent bonding at phosphorus is best described as a single highly polarized σ bond.^36^ The negative charge in **1**^**–**^ is likely delocalized (Figure 1B), and herein, double bonds are drawn for the sake of simplicity to track valence, per IUPAC recommendations.^37^ Rather than formal bond order, a qualitative variation of the P–O or P–N interaction (and relative charge localization) is observed in SC-XRD data by comparing bond distances. For example, when **1**^**–**^ binds κ_1_ through oxygen in **3-La**, the P–N^1°^ length is much shorter [1.560(1) Å] than the average P–N^1°^ distance [1.606(2) Å] of the two ligands bound in a κ^2^ mode (Table 1). The lanthanum-bound P–O distance [1.549(1) Å] is also longer than the average of the P–O distances [1.526(1) Å] for the ligands bound in κ^2^ mode .

**Figure 4:**
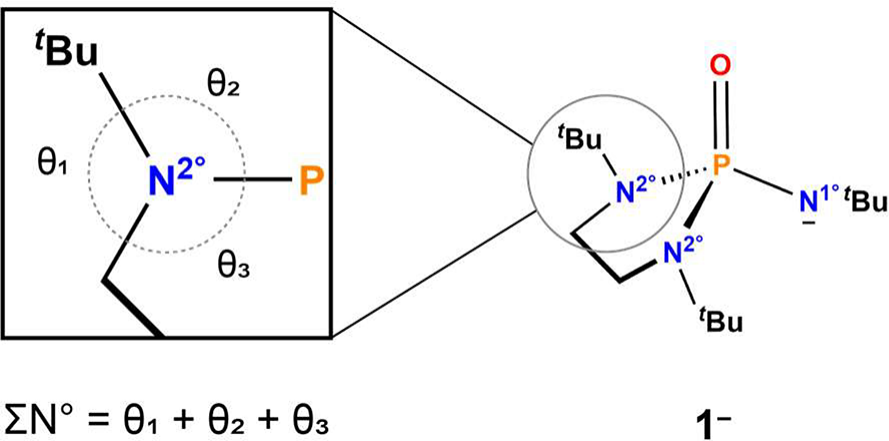
Visual depiction of ∑N° input angles.

The structural characterization^38^ of (Me_2_N)PF_2_ revealed an (at the time) unexpected planar geometry at N and a shorter-than-expected P–N distance of 1.628(5) Å. Subsequent investigations noted the exceptional electron richness that dialkylamido substituents confer to phosphines due to π donation of the N lone pair to the phosphorus center.^39−41^ This interaction has also been identified in pentavalent phosphorus systems^42,43^ and contributes to the strong stabilization of high-valence *f*-element species^26,27,44,45^ by dialkylamido imidophosphorane ligands. The zwitterionic character of (NR_2_)_3_P=E (E = O, S, or N^–^) is enhanced by donation from the dialkylamides. The planarity (or pyramidal distortion) at the nitrogen can be quantified in the solid-state structures,^42,44^ indicating which N atoms are participating in π donation to phosphorus. The planarity at N^2°^ and P–N^2°^ bond distances can shed light on the delocalization of electron density across the ligand structure. A sum of angles around N^2°^ (∑N°) quantifies the planarity of the N^2°^ geometry and is defined as the sum of the three angles formed between N and C/P (Figure 4). The pyramidalization of N in such phosphorus moieties, particularly when coordinated to a metal, can provide information about the degree of electron donation to the P center.^44,45^

A ∑N° value close to 360° indicates planarity, while deviation from 360°, generally to around 350° herein, indicates a more pyramidal geometry. On the basis of these criteria, the N^2°^ moieties can be sorted as planar, denoted as N^2°†^ (∑N° > 355°), and pyramidal, denoted as N^2°Δ^ (∑N° < 355°). With the electron-withdrawing Cl^–^ substituent, both N^2°^ atoms of the diethylamide backbone in **1-Cl** display similar planarity [∑N° = 355(1)°] and short P–N^2°^ bond lengths [1.634(4) Å], indicating substantial π donation to P. Installation of the N^1°^H^*t*^Bu substituent marks a clear distinction between the two backbone N^2°^ atoms, resulting in one more planar N^2°†^ [∑N° = 355.1(2)°] and one more pyramidal N^2°Δ^ [∑N° = 350.1(2)° (Table 1)]. The lengthening of the average P–N^2°^ backbone distance is observed [1.674(2) Å in **1-H** vs 1.634(4) Å in **1-Cl** (Table 1)], consistent with a weaker P–N^2°^ π interaction when the electron-withdrawing Cl^–^ is replaced with the -N^1°^H^*t*^Bu moiety.

Deprotonation of **1-H** to **1-K** effects starker differences in the geometries of N^2°^. The structure of **1-K** exhibits two distinct ligand moieties in the asymmetric unit; one acts as a bridge to another crystallographically identical unit to form the tetrameric configuration in the solid state (Figure 3). The terminal ligand unit reveals one planar N^2°^ geometry [∑N°^†^ = 359.1(1)°] and one more pyramidal N^2°^ [∑N°^Δ^ = 350.4(2)°]. The difference in the degree of π donation suggested by the ∑N° values is also reflected in the P–N^2°^ distances; the P–N^2°†^ distance [1.693(2) Å] is slightly shorter than the P–N^2°Δ^ distance [1.706(1) Å]. One of the backbone N^2°^ moieties in the bridging ligand in **1-K** also coordinates to K^+^, resulting in a significantly distorted geometry, with ∑N° = 349.6(2)°. This metric is excluded in the averages presented in Table 1, as coordination at the backbone N^2°^ is unique to the structure of **1-K**.

The ∑N°^†^ values observed herein are similar to those reported in the literature, which often range between 356° and 360°.^39,44^ The pyramidally distorted N centers generally display ∑N°^Δ^ values close to 350°, which is closer to planar than other examples in the literature, which are reported to be as low as 338(2)°.^44^ This is expected due to the geometry constraints enforced by the chelating nature of the backbone, which encourages more planar geometries at N. This increased planarity was shown to facilitate π donation to P in a related imidophosphorane ligand, increasing the basicity and electron-donating capability of the ligand.^26^

The deprotonated ligand binds to metal centers in both monodentate (κ^1^) and bidentate (κ^2^) modes. There is generally a preference for the chelating, κ^2^ mode, which is common among anionic phosphoramide ligands.^29,46−56^ The steric bulk of the *tert*-butyl groups prevents the formation of multimetallic species by serving as a bridge, which is sometimes observed for similar anionic phosphoramides.^46,50,51,57−59^ The only instance in which **1**^**–**^ acts as a bridge is in the structure of **1-K** (Figure 3). The κ^1^ mode is mainly observed for protonated forms of the ligand in structures of **4-LaI** and **4-La-pF** (Figure 2E,F); however, one anionic ligand is bound in a κ^1^ mode to lanthanum in the structure of **3-La** (Figure 2D). When protonated, the ligand is observed to participate in hydrogen bonding interactions between the protonated ligand’s N–H and an oxygen atom on a neighboring ligand in the structures of **4-LaI** and **4-La-pF**. The O–N distances are relatively short between the pair participating in hydrogen bonding (O···H–N), 2.860(5) Å for **4-LaI** and shorter, 2.815(2) Å, for **4-La-pF**. This hydrogen bonding interaction may play a part in maintaining the structural integrity in **4-LaI** and **4-La-pF**, rather than ejecting a neutral equivalent of **1-H** from the coordination sphere. The oxophilic nature of La^3+^ and zwitterionic character at oxygen must also be considered.

In complexes **2-LaI**, **2-LaBn**, and **2-La-pF** in which both ligands are anionic and coordinated in bidentate mode, N planarity metrics are similar to those of **1-H** and **1-K**, where one N^2°^ is slightly more planar than the other. This trend is also observed in the bidentate mode, anionic ligand moieties in **3-La**, **4-LaI**, and **4-La-pF**. The only case in which both N^2°^ moieties are relatively planar is in **1-Cl**, where an intermediate ∑N° value of 355(1)° is observed (Table 1). The SC-XRD structure of **3-La** showcases the variable nature of the interactions between P and O and N^1°^. In other words, as the steric and electrostatic demands at the coordination site vary, the ligand responds with modulation of the P–N^1°^ and P–O distances, blurring the lines between formal P–N^1°^ and P–O versus P=N^1°^ and P=O moieties. The monodentate **1**^**–**^ ligand in **3-La** exhibits a markedly shorter pendent P–N^1°^ distance of 1.560(1) Å, compared to the average of 1.606(2) Å for the other two, La-bound N^1°^ atoms. This change is also reflected in the P–O distances, with the P–O distance for the O-bound ligand being longer [1.549(1) Å] than for the bidentate ligands [1.526(1) Å].

The protonated ligands in **4-LaI** and **4-La-pF** display similar trends, with a notably shorter average κ^1^ P–O bond of 1.508(1) Å compared to those of the two anionic, κ^2^ ligands [1.532(3) Å]. The κ^1^ P–N^1°^ distance in **4-LaI** and **4-La-pF** is lengthened due to protonation and participation of hydrogen bonding, resulting in a longer average P–N^1°^ distance of 1.629(4) Å versus a distance of 1.594(5) Å for the anionic, κ^2^-bound ligands. In aggregate, these data highlight the variable P–N and P–O bonds that respond to the ligand protonation state, as well as metal coordination number and intramolecular H-bonding in the secondary coordination sphere.

The structure of **2-LaBn** incorporates a single terminal La–C σ bond; often, organometallic La complexes include multiple La–C σ bonds, though a few examples of methyl and benzyl groups have been reported.^17,60−62^ The La–CH_2_Ph distance [2.650(2) Å] is similar to those reported in the literature.^16,17,63^ The La···C_ipso_ distance [3.715 (2) Å] is sufficiently long to classify **2-LaBn** as an η^1^-benzyl complex, whereas η^2^ La···C_ipso_ distances are notably shorter, generally ∼3.0 Å.^16,17,63^

## Conclusion

A new ligand framework is introduced, which allows for deterministic synthesis of heteroleptic La^3+^ complexes featuring two or three phosphoramide ligands. The nature of the P–O and P–N bond is discussed in the context of bond lengths determined by SC-XRD and comparison to literature assignments of P=X bonds. While deprotonation occurs formally at the N–H site, there is significant charge density at the O atom. Notably, steric crowding in **3-La** demonstrates that monotopic bonding through the O atom is achievable, resulting in a short P–N distance akin to that observed in alkyl phosphinimines. The possibility of intramolecular hydrogen bonding to stabilize terminal lanthanum -oxo and -imido substituents was evaluated. However, the significant stability of **1-K** precludes the formation of anionic lanthanum complexes featuring a K^+^ cation in the complexes studied herein. The binding preferences and reaction chemistry of a new phosphoramide ligand are reported for the diamagnetic La^3+^ ion, and a variety of heteroleptic, monometallic complexes are accessible via one-step reactions.

## Experimental Details

### General Considerations

Unless otherwise noted, all manipulations were performed with rigorous exclusion of oxygen/water in an inert atmosphere glovebox (N_2_, <0.1 ppm O_2_/H_2_O) or using standard Schlenk techniques under UHP argon or nitrogen. LaI_3_(THF)_4_^64^ and benzyl potassium^65^ (KBn) were prepared according to the published procedures. Further experimental details and considerations are provided in the Supporting Information.

### Synthesis of O=P(*N*,*N*′-Di-*tert*-butylethylenediamide)Cl (**1-Cl**)

To a 500 mL Schlenk pear flask charged with 300 mL of toluene, a PTFE stir bar, and a rubber septum were added POCl_3_ (15.0 mL, 0.16 mol, 1.0 equiv), *N*,*N*′-di-*tert*-butylethylenediamine (35 mL, 0.16 mol, 1.0 equiv), and triethylamine (70 mL, 0.48 mol, 3.0 equiv) via an addition funnel under argon. The reaction mixture was stirred with heating to reflux (∼120 °C bath temperature) overnight (14 h) under argon, resulting in a brown supernatant with a colorless precipitate. Under an ambient atmosphere, the reaction mixture was filtered through Celite and the filter cake was washed with 3 × 80 mL of ethyl acetate. The volatiles were removed by rotary evaporation to give the crude product as a tan solid. The solid was sublimed at 60 °C (15 mTorr) over 7 days to give the title compound as a crystalline white solid (31.61 g, 78%) of suitable purity [∼98% by ^31^P{^1^H} NMR (Figure S5)] to proceed to the next step. Analytically pure material was obtained by recrystallization from diethyl ether. Crystals suitable for XRD analysis were grown by slow evaporation of an acetonitrile solution in air: ^1^H NMR (400 MHz, C_6_D_6_) δ 2.66–2.49 (m, 4H, C*H*_2_N^*t*^Bu), 1.25 (s, 18H, NC(C*H*_3_)_3_); ^13^C{^1^H} NMR (101 MHz, C_6_D_6_) δ 54.01 (d, *J* = 4.2 Hz, N*C*(CH_3_)_3_), 39.12 (d, *J* = 13.7 Hz, *C*H_2_N^*t*^Bu), 28.05 (d, *J* = 3.7 Hz, NC(*C*H_3_)_3_); ^31^P{^1^H} NMR (162 MHz, C_6_D_6_) δ 18.88; IR (ATR) ν 2980 (w), 2936 (w), 2860 (vw), 2732 (m), 2690 (m), 2661 (m), 2630 (m), 2610 (w), 2589 (m), 2496 (w), 2438 (m), 2391 (w), 2331 (vw), 2312 (vw), 2161 (vw), 2048 (vw), 1587 (s), 1496 (w), 1482 (w), 1451 (w), 1404 (w), 1381 (s), 1272 (vw), 1234 (m), 1195 (s), 1182 (m), 1126 (vw), 1057 (w), 1018 (m), 981 (vw), 955 (vw), 939 (vw), 916 (w), 869 (w), 744 (w), 704 (vw), 670 (vw), 654 (vw), 632 (vw), 598 (vw), 583 (vw), 523 (m), 459 (w), 423 (w) cm^–1^. Elemental analysis for C_10_H_22_ClN_2_OP, found (calcd): C, 47.79 (47.53); H, 9.01 (8.77); N, 11.13 (11.08).

### Synthesis of O=P(N,*N*′-Di-*tert*-butylethylenediamide)(NH^t^Bu) (**1-H**)

Under argon, *tert*-butyl amine (12 mL, 110 mmol, 2.4 equiv) was added via syringe to a 200 mL Schlenk pear flask charged with 100 mL of toluene and a PTFE stir bar. The solution was cooled in a dry ice/IPA bath, and then 45 mL of *n*-butyllithium (2.5 M in hexanes, 45 mL, 2.3 equiv) was added dropwise with stirring over the course of 15 min. The dry ice bath was removed, and the reaction mixture was allowed to stir for an additional 30 min, yielding a fine white suspension. In air, a 200 mL Schlenk pear flask was charged with a PTFE stir bar and **1-Cl** (11.89 g, 47 mmol, 1 equiv) and then sparged with argon for 5 min. The LiNH^*t*^Bu suspension in toluene was transferred via cannula to the flask containing **1-Cl**, and the reaction mixture was heated in an oil bath set to 100 °C overnight (14 h) with stirring under argon. The flask was then cooled in an ice bath and opened to the ambient atmosphere, and its contents were stirred for 1 h. The volatiles were removed *in vacuo*, and then 100 mL of chilled deionized water was added slowly (note that slow addition is important due to residual LiNH^*t*^Bu). The slurry was stirred vigorously for 1 h, and the precipitate was collected on a frit. The crude material was washed with an additional 50 mL of water. The tan solid was dissolved in 200 mL of petroleum ether, dried over anhydrous MgSO_4_, and then filtered through a frit packed with Celite. The volume of the filtrate was reduced *in vacuo* to approximately 150 mL in a round-bottom flask and placed in a −78 °C freezer. Colorless crystals grew overnight, and the pale-yellow supernatant was decanted off. The residual volatiles were removed *in vacuo* to give the title compound as an air-stable, crystalline white solid (10.58 g, 78%). Crystals suitable for XRD analysis were grown by room-temperature evaporation of a petroleum ether solution under the ambient atmosphere: ^1^H NMR (400 MHz, CDCl_3_) δ 3.22–2.98 (m, 4H, C*H*_2_N^*t*^Bu), 2.20 (d, *J* = 7.9 Hz, 1H, N*H*^*t*^Bu), 1.35 (d, *J* = 1.1 Hz, 18H, NC(C*H*_3_)_3_), 1.26 (d, *J* = 0.9 Hz, 9H, NHC(C*H*_3_)_3_); ^13^C{^1^H} NMR (101 MHz, CDCl_3_) δ 53.38 (d, *J* = 4.6 Hz, N*C*(CH_3_)_3_), 50.58 (d, *J* = 2.9 Hz, N*C*(CH_3_)_3_), 39.80 (d, *J* = 14.6 Hz, *C*H_2_N^*t*^Bu), 31.82 (d, *J* = 4.1 Hz, NC(*C*H_3_)_3_), 28.40 (d, *J* = 3.2 Hz, NC(*C*H_3_)_3_); ^31^P{^1^H} NMR (162 MHz, CDCl_3_) δ 15.64 (s); IR (ATR) ν 3190 (br), 3015 (vw), 2963 (m), 2926 (m), 2863 (m), 2162 (vw), 1487 (w), 1476 (w), 1439 (w), 1383 (m), 1359 (m), 1241 (m), 1207 (s), 1168 (s), 1143 (m), 1096 (w), 1061 (m), 1042 (s), 978 (m), 926 (vw), 910 (vw), 862 (m), 805 (m), 755 (w), 698 (m), 654 (m), 627 (w), 598 (w), 563 (m), 502 (m), 451 (m), 421 (vw) cm^–1^. Elemental analysis for C_14_H_32_N_3_OP, found (calcd): C, 58.23 (58.10); H, 10.91 (11.15); N, 14.64 (14.52).

### Synthesis of [K(THF)_*x*_][O=P(*N*,*N*′-Di-*tert*-butylethylenediamide)(N^*t*^Bu)] (**1-K**_**THF**_)

Inside a glovebox, **1-H** (0.684 g, 2.4 mmol) was dissolved in 4 mL of toluene in a 20 mL scintillation vial charged with a PTFE stir bar. Benzyl potassium (0.312 g, 2.4 mmol) was added as a solid, and the reaction mixture was allowed to stir at room temperature for 3 h, during which time a white precipitate formed. Three milliliters of *n*-pentane was added, and the precipitate was collected on a fine porosity frit. The white powder was washed with 2 × 5 mL of *n*-pentane, dissolved in 12 mL of THF, concentrated to a volume of 7 mL *in vacuo*, and placed in a −35 °C freezer overnight. The supernatant was decanted off of colorless crystals, and the residual volatiles were removed *in vacuo* to give the title compound as a white powder (0.691 g, 89% yield). XRD quality crystals were grown from slow diffusion of *n*-pentane into a concentrated solution in toluene at −35 °C. Note that the THF content depends on absolute vacuum and can be quantified by ^1^H NMR: ^1^H NMR (400 MHz, THF-*d*_8_) δ 3.72 (m, 1H, THF), 2.94 (s, 2H, C*H*_2_N^*t*^Bu), 2.92 (s, 2H, C*H*_2_N^*t*^Bu), 1.77 (m, 1H, THF), 1.30 (s, 18H, NC(C*H*_3_)_3_), 1.17 (d, *J* = 1.1 Hz, 9H, KNC(C*H*_3_)_3_); ^13^C{^1^H} NMR (101 MHz, THF-*d*_8_) δ 52.42 (s, N*C*(CH_3_)_3_), 50.67 (s, N*C*(CH_3_)_3_), 42.00 (s, *C*H_2_N^*t*^Bu), 35.87 (d, *J* = 12.5 Hz, NC(*C*H_3_)_3_), 29.49 (s, NC(*C*H_3_)_3_); ^31^P{^1^H} NMR (162 MHz, THF-*d*_8_) δ 7.56 (br); IR (ATR) ν 2960 (m), 2867 (w), 2855 (w), 2811 (vw), 1472 (vw), 1458 (vw), 1387 (w), 1376 (w), 1349 (m), 1251 (s), 1228 (m), 1207 (s), 1163 (m), 1142 (m), 1092 (w), 1059 (s), 1015 (m), 971 (m), 864 (w), 837 (m), 793 (m), 716 (m), 626 (m), 596 (w), 559 (w), 507 (s), 484 (m), 424 (vw), 401 (vw) cm^–1^. Elemental analysis for C_14_H_31_KN_3_OP·(C_4_H_8_O)_0.25_, found (calcd): C, 52.14 (52.86); H, 9.63 (9.70); N, 11.97 (12.16). Note that the elemental composition was calculated for 0.25 equiv of THF based on ^1^H NMR. The level of carbon is slightly low, likely due to incomplete combustion or THF loss.

### Synthesis of [K][O=P(*N*,*N*′-Di-*tert*-butylethylenediamide)(N^t^Bu)] (**1-K**)

Inside a glovebox, a 1 L round-bottom flask was charged with 300 mL of diethyl ether, potassium hydride (1.76 g, 44 mmol, 1.2 equiv), and a PTFE stir bar and then sealed with a rubber septum. The flask was removed from the glovebox and cycled onto a Schlenk line. **1-H** (10.58 g, 40 mmol, 1.0 equiv) was added as a solid against a positive flow of nitrogen gas. The reaction mixture was stirred overnight (16 h) with the vessel open to nitrogen flow to relieve pressure and then filtered through a Schlenk frit packed with Celite. An additional 150 mL of diethyl ether was used to wash the filter cake, then the volatiles were removed from the filtrate *in vacuo* for 48 h to give the title compound as a white powder (10.32 g, 86%): ^1^H NMR (400 MHz, THF-*d*_8_) δ 2.97–2.90 (m, 4H, C*H*_2_N^*t*^Bu), 1.30 (s, 18H, NC(C*H*_3_)_3_), 1.18 (s, 9H, NC(C*H*_3_)_3_); ^13^C{^1^H} NMR (101 MHz, THF-*d*_8_) δ 51.46 (s, N*C*(CH_3_)_3_), 49.55 (d, *J* = 4.8 Hz, N*C*(CH_3_)_3_), 40.64 (d, *J* = 9.5 Hz, *C*H_2_N^*t*^Bu), 34.24 (s, NC(*C*H_3_)_3_), 28.23 (d, *J* = 2.9 Hz, NC(*C*H_3_)_3_); ^31^P{^1^H} NMR (162 MHz, THF-*d*_8_) δ 8.30 (br).

### Synthesis of La(O=P(*N*,*N*′-Di-*tert*-butylethylenediamide)(N^t^Bu))_2_(DMAP)I (**2-LaI**)

LaI_3_(THF)_4_ (137 mg, 0.17 mmol, 1 equiv), **1-K** (111 mg, 0.34 mmol, 2 equiv), and DMAP (21 mg, 0.17 mmol, 1 equiv) were combined as solids in a 20 mL scintillation vial charged with a PTFE stir bar and then dissolved in 4 mL of diethyl ether. One milliliter of diethyl ether was used to rinse any remaining starting materials into the reaction vial, and the reaction mixture stirred for 14 h at room temperature. The crude mixture was filtered through Celite to remove a fine white precipitate, and the colorless filtrate was concentrated to 1 mL and placed in a −35 °C freezer overnight. The supernatant was decanted from colorless crystals, and the residual volatiles were removed *in vacuo* to give the title compound as a crystalline white solid (75 mg, 46%): ^1^H NMR (400 MHz, C_6_D_6_) δ 9.23 (d, *J* = 6.2 Hz, 2H, NC_5_*H*_4_NMe_2_), 5.89 (d, *J* = 6.2 Hz, 2H, NC_5_*H*_4_NMe_2_), 2.87–2.72 (m, 8H, C*H*_2_N^*t*^Bu), 2.01 (s, 6H, NC_5_H_4_N(C*H*_3_)_2_), 1.67 (s, 18H, NC(C*H*_3_)_3_), 1.53 (s, 18H, NC(C*H*_3_)_3_), 1.38 (s, 18H, NC(C*H*_3_)_3_); ^13^C{^1^H} NMR (101 MHz, C_6_D_6_) δ 150.89 (s, N*C*_5_H_4_NMe_2_), 105.76 (s, N*C*_5_H_4_NMe_2_), 53.62 (s, N*C*(CH_3_)_3_), 52.13 (s, N*C*(CH_3_)_3_), 52.07 (s, N*C*(CH_3_)_3_), 40.56 (s, *C*H_2_N^*t*^Bu), 38.05 (s, NC_5_H_4_N(C*H*_3_)_2_), 34.44 (d, *J* = 10.9 Hz, NC(*C*H_3_)_3_), 29.54 (s, NC(*C*H_3_)_3_), 29.21 (s, NC(*C*H_3_)_3_) (note that in this complex the backbone N^*t*^Bu groups are inequivalent); ^31^P{^1^H} NMR (162 MHz, C_6_D_6_) δ 18.06 (s); IR (ATR) ν 3017 (vw), 2969 (m), 2935 (w), 2862 (w), 1618 (m), 1535 (w), 1475 (vw), 1449 (w), 1386 (m), 1358 (m), 1269 (w), 1250 (m), 1205 (s), 1141 (m), 1120 (m), 1033 (s), 998 (s), 982 (m), 948 (w), 908 (vw), 867 (w), 842 (s), 803 (m), 740 (s), 649 (m), 633 (m), 600 (w), 579 (m), 529 (s), 517 (s), 461 (vw), 433 (w), 406 (w) cm^–1^. Elemental analysis for C_35_H_72_ILaN_8_O_2_P_2_, found (calcd): C, 43.60 (43.57); H, 7.54 (7.52); N, 11.55 (11.61).

### Synthesis of La(O=P(N,*N*′-Di-*tert*-butylethylenediamide)(N^t^Bu))_2_(THF)(CH_2_C_6_H_5_) (**2-LaBn**)

Inside a glovebox, **1-K** (104 mg, 0.32 mmol, 2 equiv) and benzyl potassium (21.0 mg, 0.16 mmol, 1.01 equiv) were added as solids to a 20 mL scintillation vial charged with LaI_3_(THF)_4_ (128 mg, 0.16 mmol, 1.0 equiv), 4 mL of toluene, and a PTFE stir bar. After being stirred overnight (14 h), the colorless slurry was filtered through a M porosity frit packed with Celite. The filter cake was washed with 3 × 1 mL of toluene, and then the filtrate was concentrated to ∼1.5 mL *in vacuo*. The solution was filtered through a pipet filter into a vial, layered with 2 mL of *n*-pentane, and then placed in a −35 °C. After 3 days, the supernatant was removed from colorless crystals and the residual volatiles were removed *in vacuo* to give the title compound as a crystalline white solid (78 mg, 56%): ^1^H NMR (400 MHz, C_6_D_6_) δ 7.28 (t, *J* = 7.3 Hz, 2H, CH_2_(C_6_*H*_5_)), 7.19 (m, 2H, CH_2_(C_6_*H*_5_)), 6.73 (t, *J* = 6.9 Hz, 1H, CH_2_(C_6_*H*_5_)), 2.84–2.71 (m, 8H), 2.20 (s, 2H, C*H*_2_(C_6_H_5_)), 1.48 (s, 18H, NC(C*H*_3_)_3_), 1.38 (s, 36H, NC(C*H*_3_)_3_); ^13^C{^1^H} NMR (101 MHz, C_6_D_6_) δ 123.12 (s, CH2(*C*_6_H_5_)), 53.48 (d, *J* = 3.7 Hz, N*C*(CH_3_)_3_), 51.86 (d, *J* = 5.8 Hz, N*C*(CH_3_)_3_), 40.78 (d, *J* = 11.6 Hz, *C*H_2_N^*t*^Bu), 34.04 (d, *J* = 11.1 Hz, NC(*C*H_3_)_3_), 30.66 (s, *C*H_2_(C_6_H_5_)), 29.35 (s, NC(*C*H_3_)_3_) (note that overlap with C_6_D_6_ precludes identification of all arene ^13^C shifts); ^31^P{^1^H} NMR (162 MHz, C_6_D_6_) δ 17.49 (s); IR (ATR) ν 2963 (s), 2859 (m), 1475 (w), 1385 (w), 1359 (m), 1274 (m), 1251 (m), 1209 (s), 1143 (m), 1127 (m), 1076 (m), 1048 (s), 980 (w), 838 (m), 801 (w), 782 (vw), 735 (m), 639 (w), 598 (vw), 571 (vw), 516 (m), 499 (m), 435 (vw) cm^–1^. Elemental analysis for C_39_H_76_LaN_6_O_3_P_2_, found (calcd): C, 53.41 (53.36); H, 8.84 (8.73); N, 9.45 (9.57).

### Synthesis of La(O=P(*N*,*N*′-Di-*tert*-butylethylenediamide)(N^t^Bu))_3_(THF)_*x*_ (**3-La**)

Inside a glovebox, LaI_3_(THF)_4_ (0.859 g, 1.1 mmol, 1.0 equiv) and **1-K** (1.04 g, 3.2 mmol, 3.0 equiv) were added to a 20 mL scintillation vial charged with a PTFE stir bar and 10 mL of diethyl ether. The reaction mixture was allowed to stir overnight at room temperature and then filtered through a M porosity frit packed with Celite. The filter cake was washed with diethyl ether (3 × 5 mL), and then the volatiles were removed from the colorless filtrate *in vacuo*. The residue was triturated with 3 × 1 mL of *n*-pentane, taken up in 5 mL of THF, and filtered through a pipet filter packed with Celite. The filtrate was concentrated to ∼2 mL and then placed in a −35 °C freezer overnight. The supernatant was decanted from colorless crystals, and the volatiles were removed *in vacuo* to give the title compound as a white powder (0.925 g, 80%). Note that in addition to one coordinated THF molecule, three THF molecules are found in the crystal lattice. The amount of THF observed by ^1^H NMR is consistent with elemental analysis and is dependent on absolute vacuum: ^1^H NMR (400 MHz, C_6_D_6_) δ 3.58 (m, 2H, THF), 2.92–2.82 (m, 12H, C*H*_2_N^*t*^Bu), 1.65 (d, *J* = 1.1 Hz, 27H, NC(C*H*_3_)_3_), 1.49 (s, 54H, NC(C*H*_3_)_3_), 1.42 (m, 2H, THF); ^13^C{^1^H} NMR (101 MHz, C_6_D_6_) δ 67.84 (s, THF), 54.51 (s, N*C*(CH_3_)_3_), 51.97 (s, N*C*(CH_3_)_3_), 41.79 (d, *J* = 14.0 Hz, *C*H_2_N^*t*^Bu), 34.19 (d, *J* = 11.4 Hz, NC(*C*H_3_)_3_), 30.65 (s, NC(*C*H_3_)_3_), 25.81 (s, THF); ^31^P{^1^H} NMR (162 MHz, C_6_D_6_) δ 21.07 (s); IR (ATR) ν 2961 (m), 2931 (m), 2908 (m), 2858 (w), 1462 (vw), 1386 (w), 1358 (m), 1272 (m), 1249 (m), 1205 (s), 1147 (m), 1128 (m), 1110 (m), 1053 (s), 1031 (m), 970 (s), 913 (w), 893 (vw), 866 (m), 840 (m), 800 (w), 740 (m), 716 (w), 644 (m), 626 (m), 599 (w), 567 (w), 517 (m), 503 (m), 433 (w), 406 (vw) cm^–1^. Elemental analysis for C_42_H_93_LaN_3_O_3_P_3_·(C_4_H_8_O)_0.5_, found (calcd): C, 51.83 (52.21); H, 9.52 (9.47); N, 11.33 (11.66).

### Synthesis of La(O=P(*N*,*N*′-Di-*tert*-butylethylenediamide)(N^t^Bu))_2_(O=P(*N*,*N*′-Di-*tert*-butylethylenediamide)(NH^t^Bu))I·(C_7_H_8_)_0.5_ (**4-LaI**)

Inside a glovebox, **1-K** (138 mg, 0.4 mmol, 2 equiv) and **1-H** (61 mg, 0.2 mmol, 1 equiv) were dissolved in 6 mL of diethyl ether in a 20 mL scintillation vial. This solution was then added to a 20 mL scintillation vial charged with LaI_3_(THF)_4_ (170 mg, 0.2 mmol, 1 equiv) and a PTFE stir bar. The reaction mixture was allowed to stir overnight (16 h) at room temperature and then filtered through a fine porosity frit packed with Celite. The volatiles were removed *in vacuo*, and the white residue was triturated with 3 × 1 mL of *n*-pentane to give the crude product as a white powder. The powder was taken up in 2 mL of toluene and filtered through a pipet filter packed with Celite, and the volume was reduced to 1 mL *in vacuo*. The solution was layered with 3 mL of *n*-pentane and placed in a −35 °C freezer for 3 days, during which time colorless crystals grew. The supernatant was decanted off, and the residual volatiles were removed *in vacuo* to give the title compound as a crystalline white solid (198 mg, 83%): ^1^H NMR (400 MHz, C_6_D_6_) δ 7.12 (m, 1.5H, toluene), 7.02 (m, 1H, toluene), 5.40 (d, *J* = 7.7 Hz, 1H, N*H*^*t*^Bu), 2.83–2.77 (m, 10H, C*H*_2_N^*t*^Bu), 2.69–2.54 (m, 2H, C*H*_2_N^*t*^Bu), 2.11 (s, 1.5H, toluene), 1.81 (s, 18H, NC(C*H*_3_)_3_), 1.49 (s, 18H, NC(C*H*_3_)_3_), 1.46 (s, 36H, NC(C*H*_3_)_3_), 1.31 (s, 9H, NC(C*H*_3_)_3_); ^13^C{^1^H} NMR (101 MHz, C_6_D_6_) δ 129.33 (s, toluene), 128.57 (s, toluene), 125.70 (s, toluene), 54.16 (d, *J* = 4.7 Hz, N*C*(CH_3_)_3_), 52.85 (d, *J* = 6.0 Hz, N*C*(CH_3_)_3_), 51.45 (s, N*C*(CH_3_)_3_), 50.34 (s, N*C*(CH_3_)_3_), 40.84 (br, *C*H_2_N^*t*^Bu), 40.69 (s, *C*H_2_N^*t*^Bu), 35.86 (d, *J* = 10.9 Hz, NC(*C*H_3_)_3_), 32.72 (d, *J* = 4.5 Hz, NC(*C*H_3_)_3_), 30.54 (br, NC(*C*H_3_)_3_), 30.04 (br, NC(*C*H_3_)_3_); ^31^P{^1^H} NMR (162 MHz, C_6_D_6_) δ 18.62 (s, 2P), 14.40 (s, 1P); IR (ATR) ν 3202 (br), 2964 (m), 2934 (w), 2860 (w), 2159 (vw), 1474 (w), 1386 (m), 1358 (m), 1272 (m), 1249 (w), 1206 (s), 1137 (m), 1109 (m), 1093 (m), 1052 (s), 1013 (s), 981 (m), 866 (m), 844 (m), 807 (m), 734 (s), 696 (vw), 665 (w), 643 (m), 596 (w), 560 (w), 528 (m), 513 (m), 502 (m), 464 (vw), 432 (vw), 405 (w) cm^–1^. Elemental analysis for C_42_H_94_ILaN_3_O_3_P_3_·(C_7_H_8_)_0.5_, found (calcd) C, 46.36 (46.39); H, 8.69 (8.39); N, 10.43 (10.70).

### Synthesis of La(O=P(*N*,*N*′-Di-*tert*-butylethylenediamide)(N^t^Bu))_2_(O=P(*N*,*N*′-Di-*tert*-butylethylenediamide)(NH^*t*^Bu))((4-F-C_6_H_4_)NH)·THF (**4-La-pF**)

Inside a glovebox, **3-La** (369 mg, 0.3 mmol, 1.0 equiv) was dissolved in 5 mL of THF in 20 mL scintillation vial, and the solution was added to a 50 mL Schlenk pear flask equipped with a PTFE stir bar and a glass stopper. The flask was cycled onto a Schlenk line, and the solution was frozen using liquid nitrogen. Then, 1.62 mL of a 200 mM solution of *p*-fluoroaniline in THF (0.3 mmol, 1 equiv) was added dropwise to the stirring solution as it thawed. The reaction mixture was stirred while warming to room temperature for 1 h, and then the volatiles were removed *in vacuo* to yield a white residue. Inside a glovebox, the crude product was taken up in 3 mL of THF and filtered through a pipet filter. The volume was reduced to 2 mL *in vacuo*, and the sample was layered with 2 mL of HMDSO and then placed in a −35 °C freezer. After 3 days, crystals formed, the supernatant was decanted off, and the residual volatiles were removed *in vacuo* to give the title compound as a white crystalline solid (293 mg, 78%): ^1^H NMR (400 MHz, C_6_D_6_) δ 7.02 (t, *J* = 8.8 Hz, 2H, NH(C_6_*H*_4_)-F), 6.77 (m, 2H, NH(C_6_*H*_4_)-F), 5.66 (d, *J* = 8.0 Hz, 1H, N*H*^*t*^Bu), 4.62 (s, 1H, N*H*(C_6_H_4_)-F), 2.85–2.83 (m, 8H, C*H*_2_N^*t*^Bu), 2.74 (s, 2H, C*H*_2_N^*t*^Bu), 2.58 (d, *J* = 12.8 Hz, 2H), 1.76 (s, 18H, NC(C*H*_3_)_3_), 1.44 (s, 36H, NC(C*H*_3_)_3_), 1.36 (s, 18H, NC(C*H*_3_)_3_), 1.29 (s, 9H, NC(C*H*_3_)_3_); ^13^C{^1^H} NMR (101 MHz, C_6_D_6_) δ 156.19 (s, NH(*C*_6_H_4_)-F), 129.42 (s, NH(*C*_6_H_4_)-F), 116.70 (d, *J* = 6.8 Hz, NH(*C*_6_H_4_)-F), 114.93 (d, *J* = 21.2 Hz, NH(*C*_6_H_4_)-F), 52.96 (s, N*C*(CH_3_)_3_), 53.59 (s, N*C*(CH_3_)_3_), 51.94 (s, N*C*(CH_3_)_3_), 50.49 (s, N*C*(CH_3_)_3_), 40.53 (s, *C*H_2_N^*t*^Bu), 39.90 (d, *J* = 14.8 Hz, *C*H_2_N^*t*^Bu), 34.91 (d, *J* = 11.3 Hz, NC(*C*H_3_)_3_), 32.36 (s, NC(*C*H_3_)_3_), 29.37 (d, *J* = 20.1 Hz, NC(*C*H_3_)_3_), 28.71 (s, NC(*C*H_3_)_3_); ^31^P{^1^H} NMR (162 MHz, C_6_D_6_) δ 18.20 (s, 2P), 15.05 (s, 1P); IR (ATR) ν 3191 (br), 2963 (m), 2928 (w), 2865 (w), 1487 (vw), 1476 (vw), 1439 (vw), 1383 (w), 1359 (w), 1241 (m), 1207 (s), 1169 (s), 1141 (m), 1096 (w), 1062 (s), 1042 (s), 979 (m), 927 (vw), 863 (m), 807 (m), 755 (vw), 696 (w), 654 (w), 627 (vw), 598 (w), 564 (m), 502 (m), 452 (w), 423 (vw) cm^–1^. Elemental analysis for C_48_H_98_FLaN_10_O_3_P_3_·(C_4_H_8_O), found (calcd): C, 52.47 (52.65); H, 9.08 (9.01); N, 11.79 (11.81).

### Reaction of **3-La** with Benzyl Potassium

Inside a glovebox, **3-La** (65 mg, 60 μmol, 1.0 equiv) was added to a 20 mL scintillation vial charged with a PTFE stir bar and dissolved in 1 mL of THF, and benzyl potassium (8 mg, 60 μmol, 1.0 equiv) was added to the reaction vial as a solution in 1 mL of THF. The orange color of benzyl potassium was bleached within a few seconds, and the reaction mixture was allowed to stir at room temperature for 10 min. NMR analysis of the crude reaction mixture indicates the formation of multiple species, including **1-K**, as well as **3-La** (Figures S32 and S33). Crystallization of the crude product from toluene at −35 °C yielded a mixture of colorless crystals that were identified as **1-K** and **2-LaBn** by SC-XRD.

### Attempted Deprotonation of **4-La-pF**

Inside a glovebox, **4-La-pF** (31.8 mg, 30 μmol, 1.0 equiv) was dissolved in 2 mL of diethyl ether in a 20 mL scintillation vial. Benzyl potassium (4 mg, 30 μmol, 1.0 equiv) was massed in a 20 mL scintillation vial, and the vial then charged with a PTFE stir bar and 1 mL of diethyl ether. The solution of **4-La-pF** was added slowly to the stirring slurry of benzyl potassium, resulting in a colorless solution. After 5 min, the reaction mixture was filtered through a pipet filter packed with Celite, concentrated to ∼1 mL, and placed in a −35 °C freezer overnight. Colorless crystals grew, which were determined to be **2-La-pF** and **1-K** by SC-XRD.

## Supporting Information Available

Further experimental considerations, NMR spectra, and crystallographic data (PDF)
