# Supplementary material for: Coordination Modes and Binding Patterns in Lanthanum Phosphoramide Complexes
Source: Inorg Chem. 2024 Mar 6;63(21):9638–47. doi: 10.1021/acs.inorgchem.3c04521 (PMC11134493; doi:10.1021/acs.inorgchem.3c04521)
Supplement: Supplementary file 1 — ic3c04521_si_001.pdf [file ic3c04521_si_001.pdf]

Supporting Information for

Coordination Modes and Binding Patterns in Lanthanum Phosphoramide Complexes

Andrew C. Boggiano†, Maximilian G. Bernbeck†, Ningxin Jiang†, Henry S. La Pierre†‡*

†School of Chemistry and Biochemistry, Georgia Institute of Technology, Atlanta, Georgia 30332-0400, United States

‡Nuclear and Radiological Engineering and Medical Physics Program, School of Mechanical Engineering, Georgia Institute of Technology, Atlanta, Georgia 30332-0400, United States

*email: hsl@gatech.edu

Table of Contents

General Considerations **2**

Nuclear Magnetic Resonance (NMR) Spectroscopy **3**

1-Cl *3*

1-H *5*

1-K$_{THF}$ *7*

1-K *8*

2-LaI *10*

2-LaBn *11*

3-La *13*

4-LaI *14*

4-La-pF *17*

Crude Reaction Mixture Between 3-La + KBn *18*

X-Ray Crystallography **23**

Refinement Details *26*

References **38**

General Considerations

Unless otherwise noted, all reagents were obtained from commercial suppliers and used as received and all manipulations were performed with rigorous exclusion of oxygen and water using Schlenk techniques under UHP argon or in an inert-atmosphere glovebox (Vigor) under a dinitrogen (<0.1 ppm O_2/H_2O) atmosphere. The glovebox is equipped with two -35 ˚C freezers and a cold well. All glassware and cannulas were stored in an oven overnight (>8h) at a temperature of ca. 160 ˚C prior to use. Celite and molecular sieves were dried under vacuum at a temperature >250 ˚C for a minimum of 24 h. Deuterated solvents were obtained from Cambridge Isotope Laboratories. C_6D_6 was stored over 3 Å molecular sieves then vacuum-transferred from purple sodium/benzophenone prior to use. THF-d_8 was degassed via 4 freeze-pump-thaw cycles and stored over 3 Å molecular sieves for a minimum of 48 h before use. *n*-Pentane, hexanes, diethyl ether, toluene, and tetrahydrofuran were purged with UHP-grade argon (Airgas) and passed through columns containing alumina/Q-5 and molecular sieves in a solvent purification system (JC Meyer Solvent Systems). 1,2-Difluorobenzene (Oakwood Chemical) was stored over 4 Å molecular sieves for 5 d, distilled under an Ar atmosphere, and stored over 3 Å molecular sieves. $POCl_3$ (Sigma-Aldrich) was distilled under argon prior to use. All solvents in the glovebox were stored in media bottles over 10% v/v 3 Å molecular sieves. Methanol was dried by refluxing over magnesium turnings activated with iodine overnight under argon and then distilled and stored over 3 Å molecular sieves. NMR spectra were obtained on a Bruker Avance III 400 MHz spectrometer at 298 K, unless otherwise noted. ^1H, ^{13}C, and ^{31}P NMR chemical shifts are reported in δ, parts per million. ^1H NMR are referenced to the residual ^1H resonances of the solvent. ^{13}C NMR are referenced to the ^{13}C resonance of the deuterated solvent. Peak position is listed, followed by peak multiplicity, integration value, and proton assignment, where applicable. Multiplicity and shape are indicated by one or more of the following abbreviations: s (singlet); d (doublet); t (triplet); q (quartet);dd (doublet of doublets); td (triplet of doublets); m (multiplet); br (broad) Elemental analyses were determined at the University of California Berkeley Microanalytical Facility (Berkeley, CA). X-ray structural determinations were performed at the Georgia Institute of Technology on a Bruker D8 Venture diffractometer.

Nuclear Magnetic Resonance (NMR) Spectroscopy

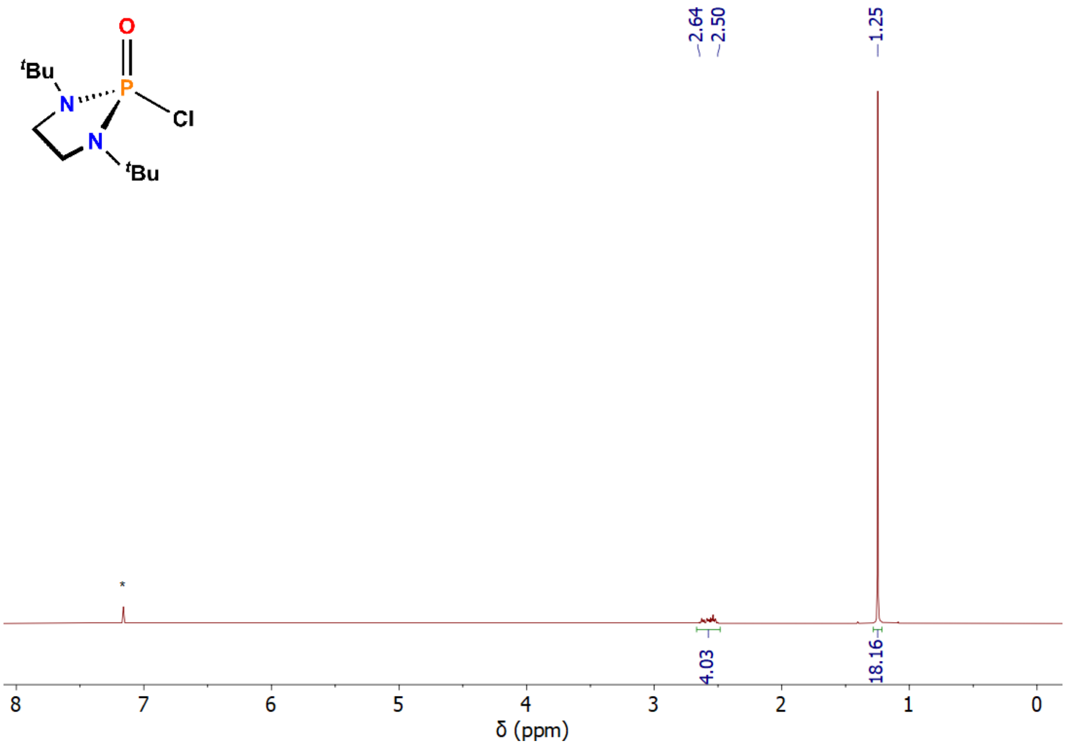

Figure S1: ^{1}H NMR of **1-Cl** in C_6D_6, C_6D_5H peak denoted by *.

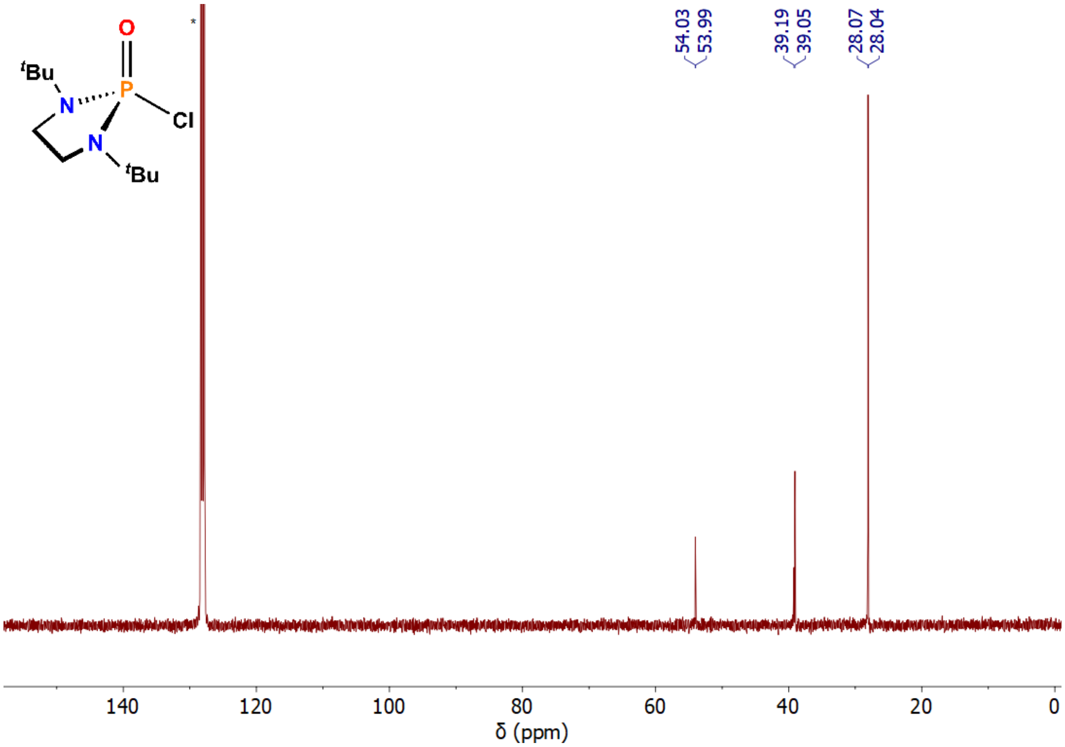

Figure S2: $^{13}C\{^{1}H\}$ NMR of **1-Cl** in C_6D_6, solvent peak denoted by *.

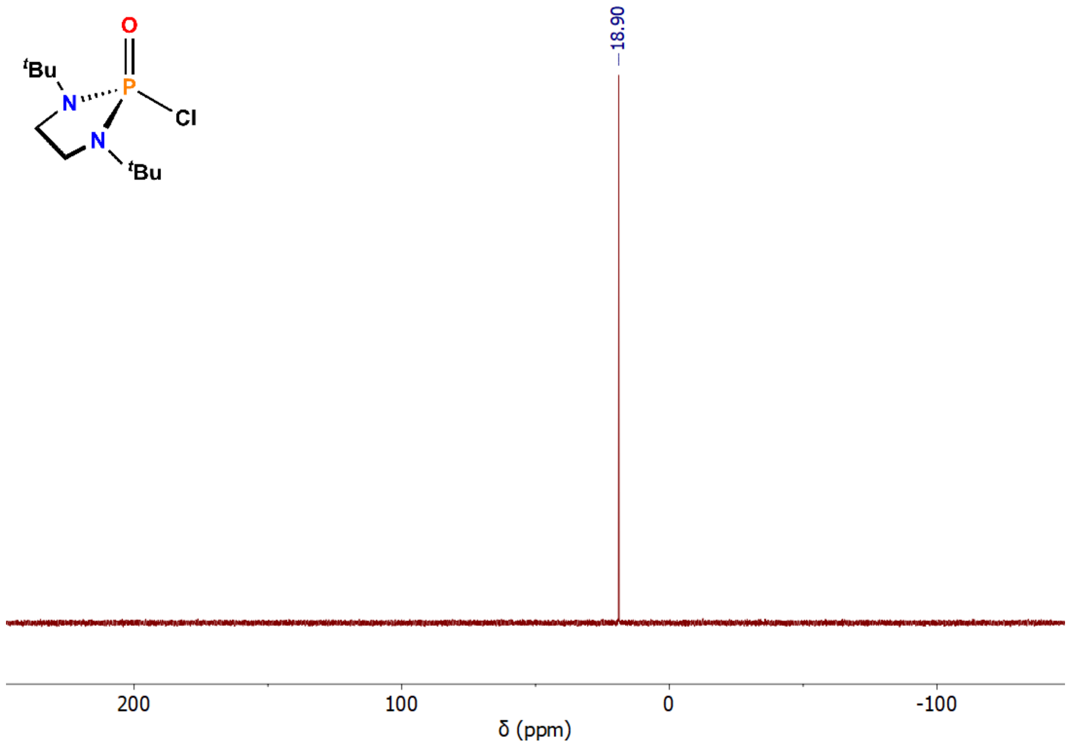

Figure S3. $^{31}P\{^{1}H\}$ NMR spectrum of **1-Cl** in C_6D_6.

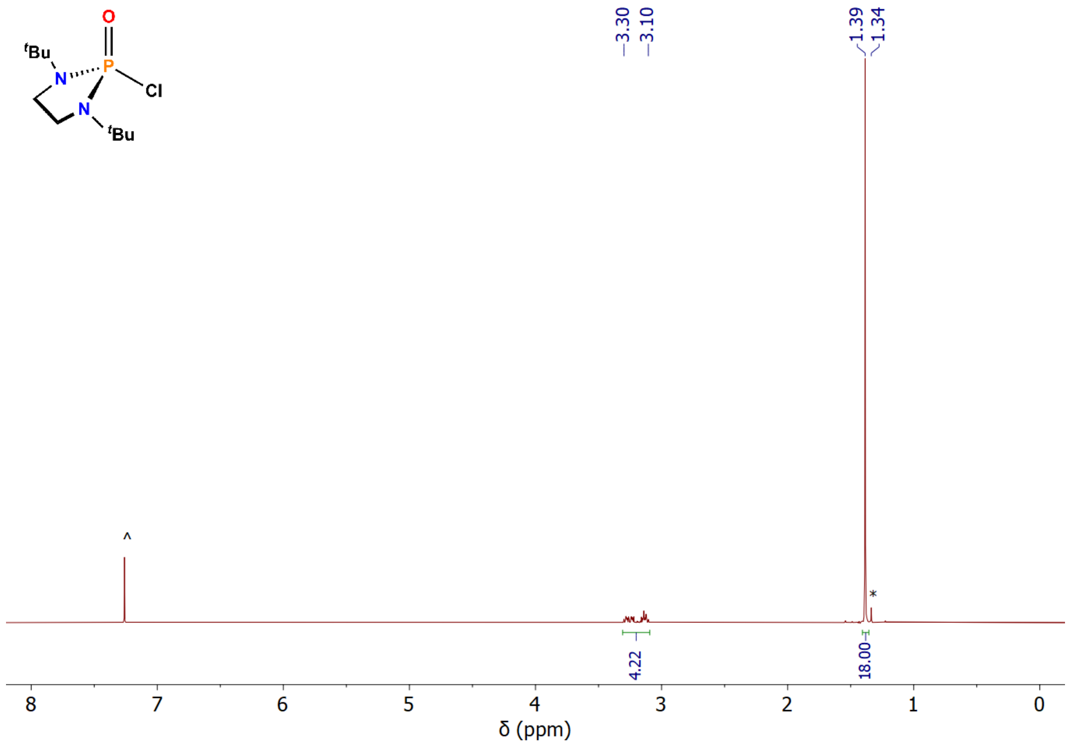

Figure S4. ^{1}H NMR spectrum of **1-Cl** after sublimation in $CDCl_3$. ^ = $CHCl_3$, * = unidentified impurity

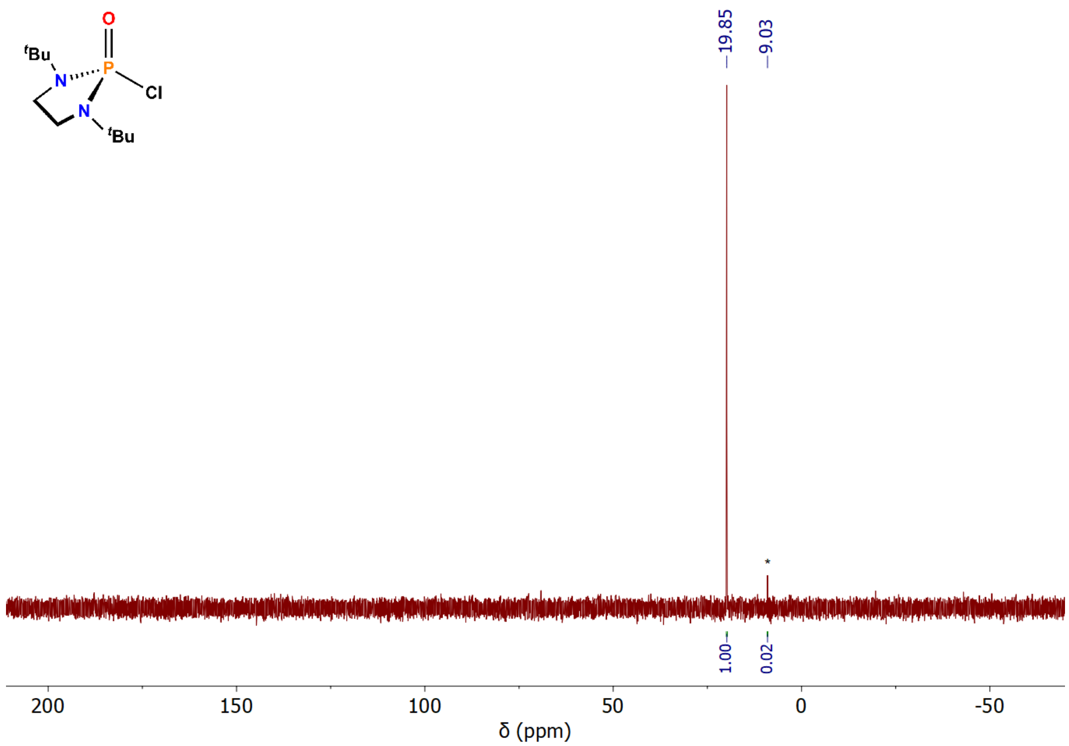

Figure S5. $^{31}P\{^{1}H\}$ NMR spectrum of **1-Cl** after sublimation in $CDCl_3$. * = unidentified impurity

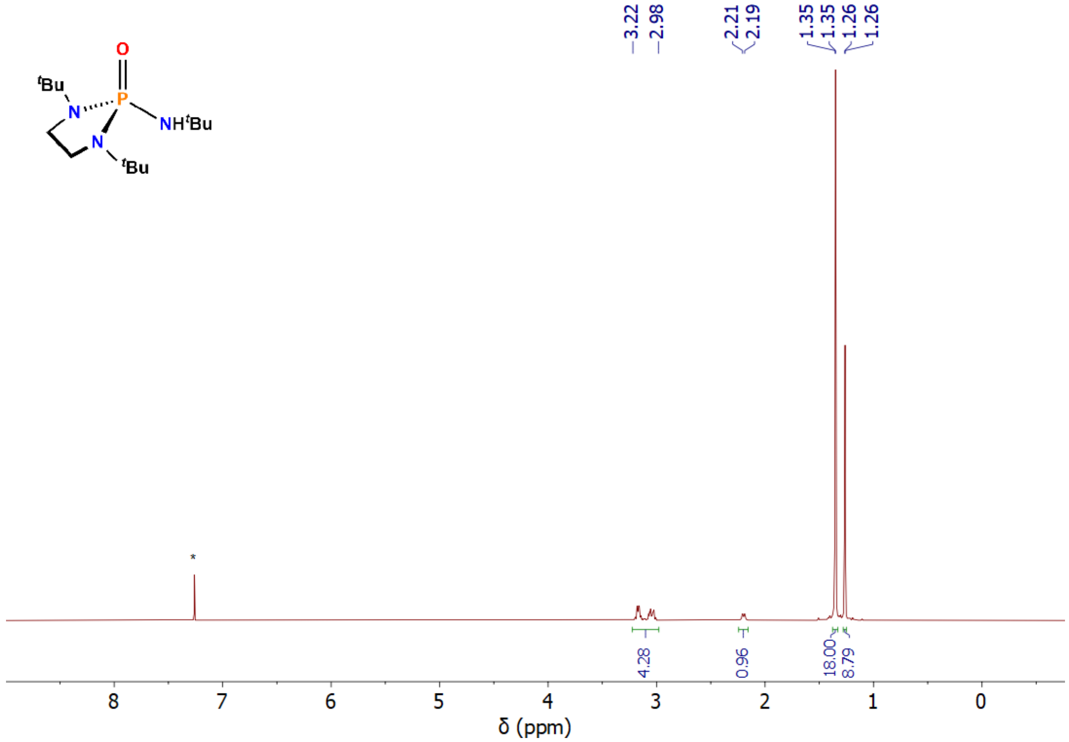

Figure S6. ^{1}H NMR spectrum of **1-H** in $CDCl_3$. * = $CHCl_3$

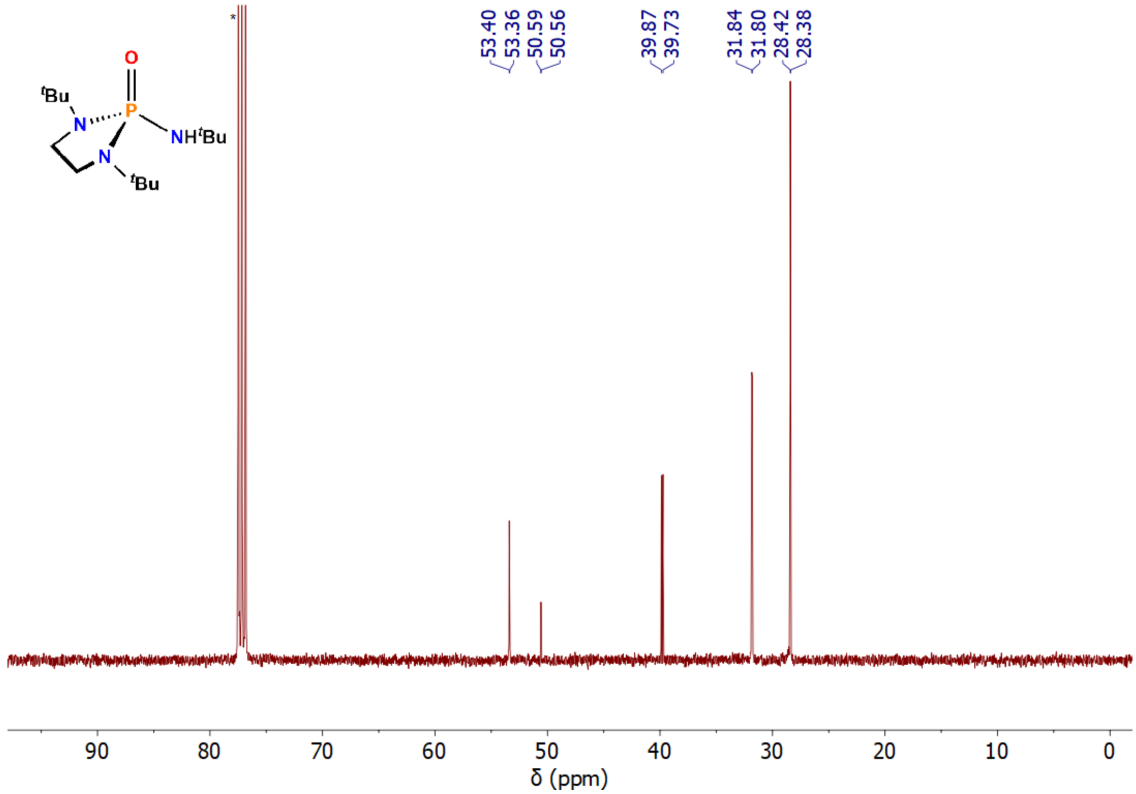

Figure S7. $^{13}C\{^{1}H\}$ NMR spectrum of **1-H** in $CDCl_3$. * = $CDCl_3$

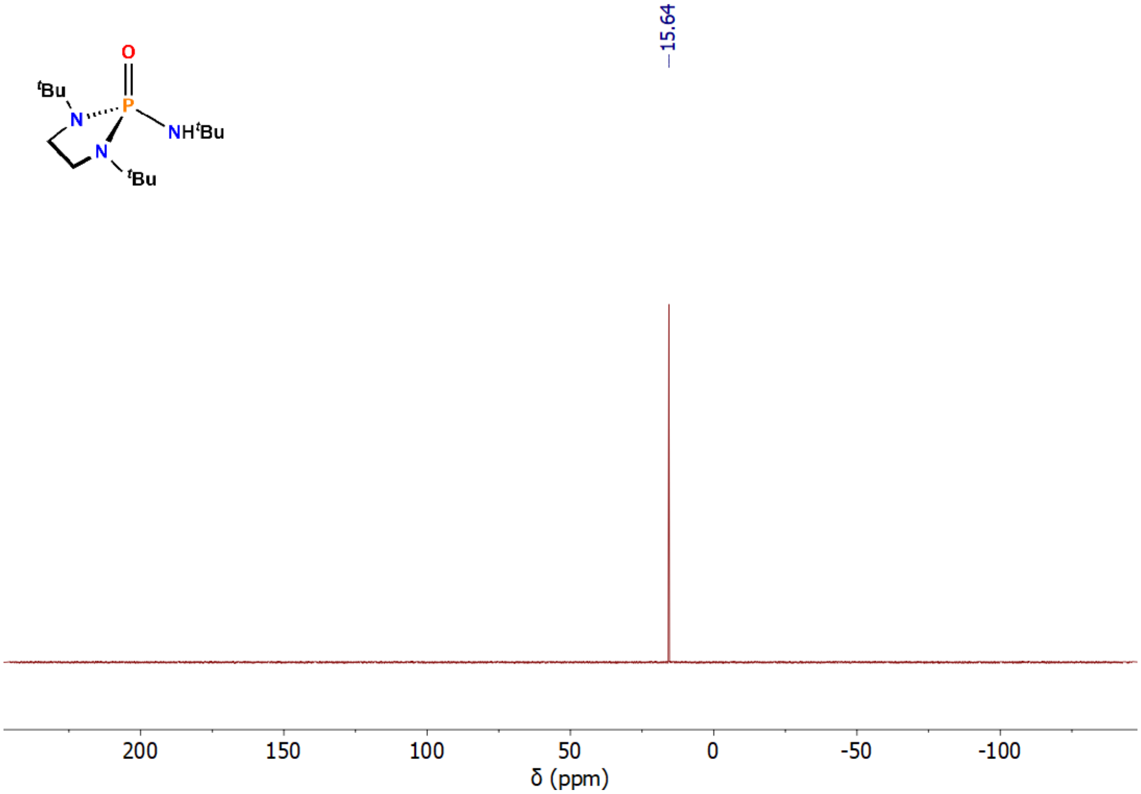

Figure S8. $^{31}P\{^{1}H\}$ NMR spectrum of **1-H** in $CDCl_3$.

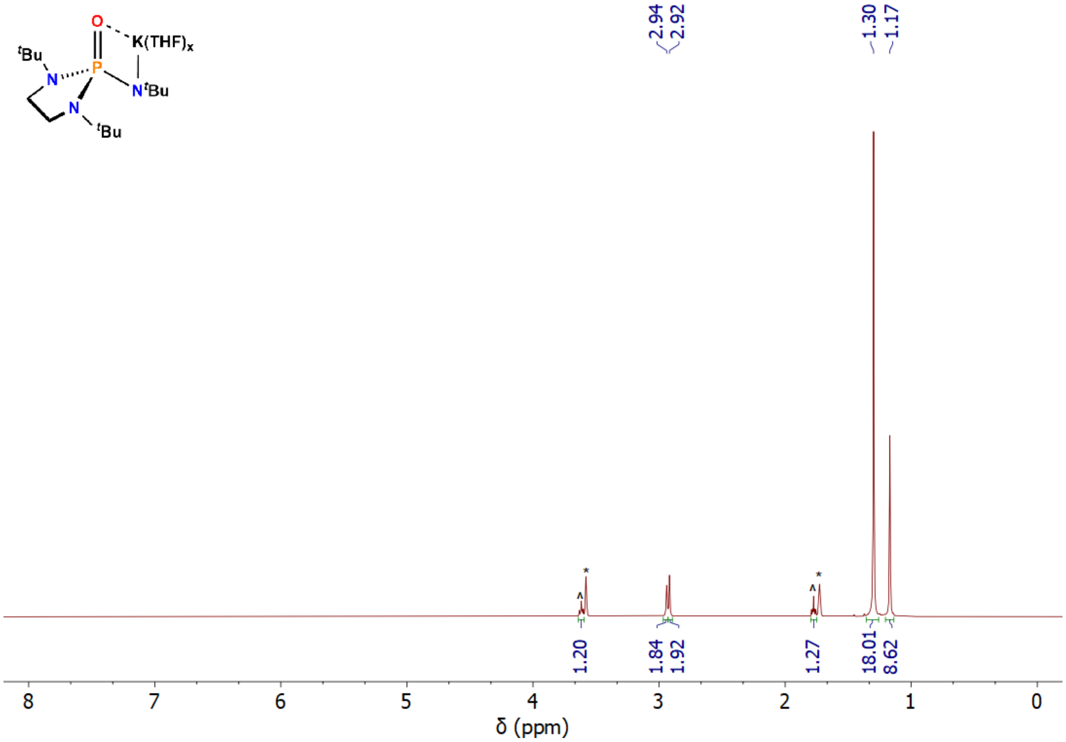

Figure S9. ^{1}H NMR spectrum of **1-K$_{THF}$** in THF-d_8. ^ = residual proteo THF, * = THF-d_7

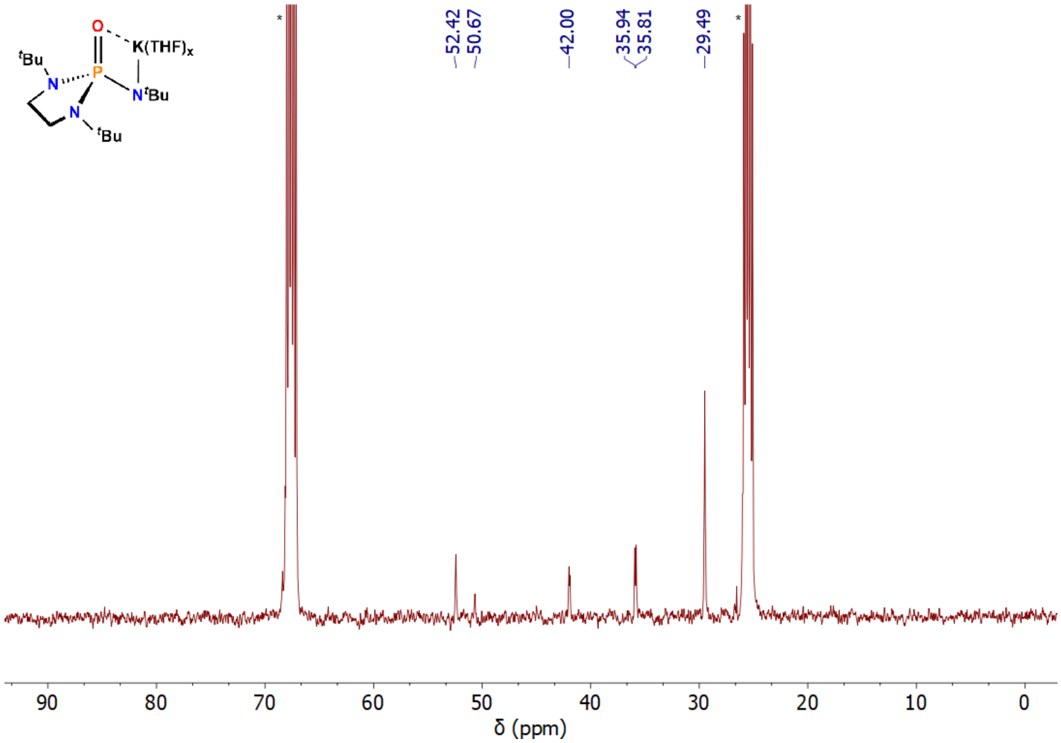

Figure S10. $^{13}C\{^{1}H\}$ NMR spectrum of **1-K$_{THF}$** in THF-d_8. * = THF-d_8

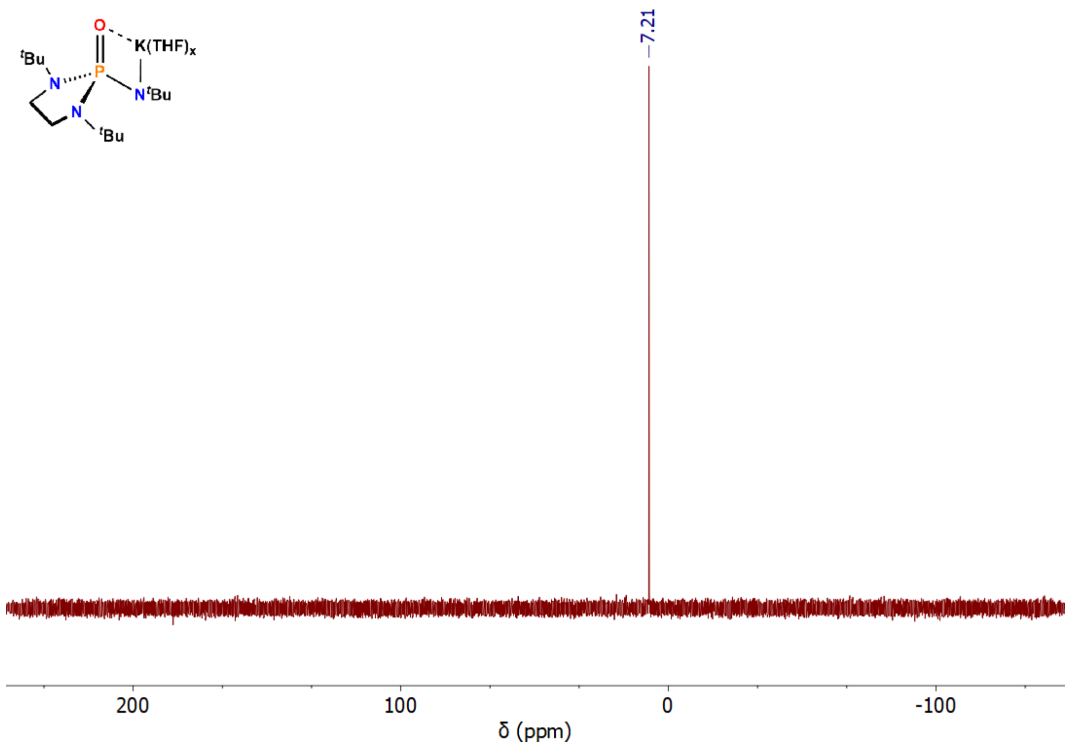

Figure S11. $^{31}P\{^{1}H\}$ NMR spectrum of **1-K$_{THF}$** in THF-d_8.

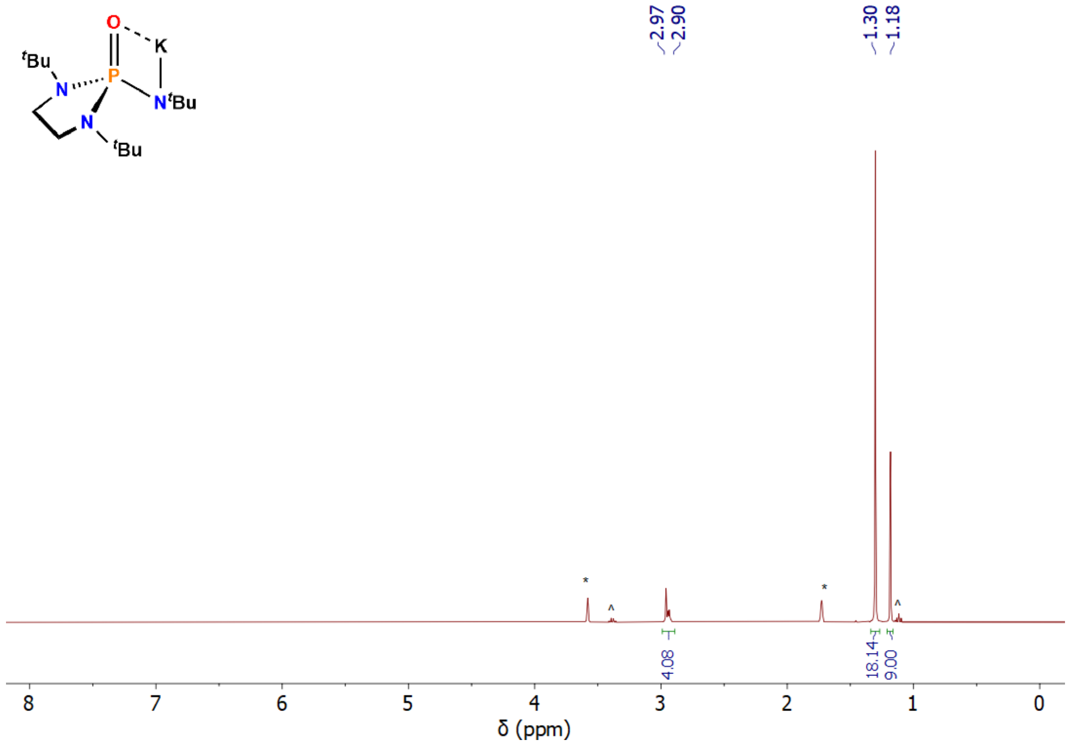

Figure S12. ^{1}H NMR spectrum of **1-K** in THF-d_8. * = THF=d_7, ^ = residual Et_2O

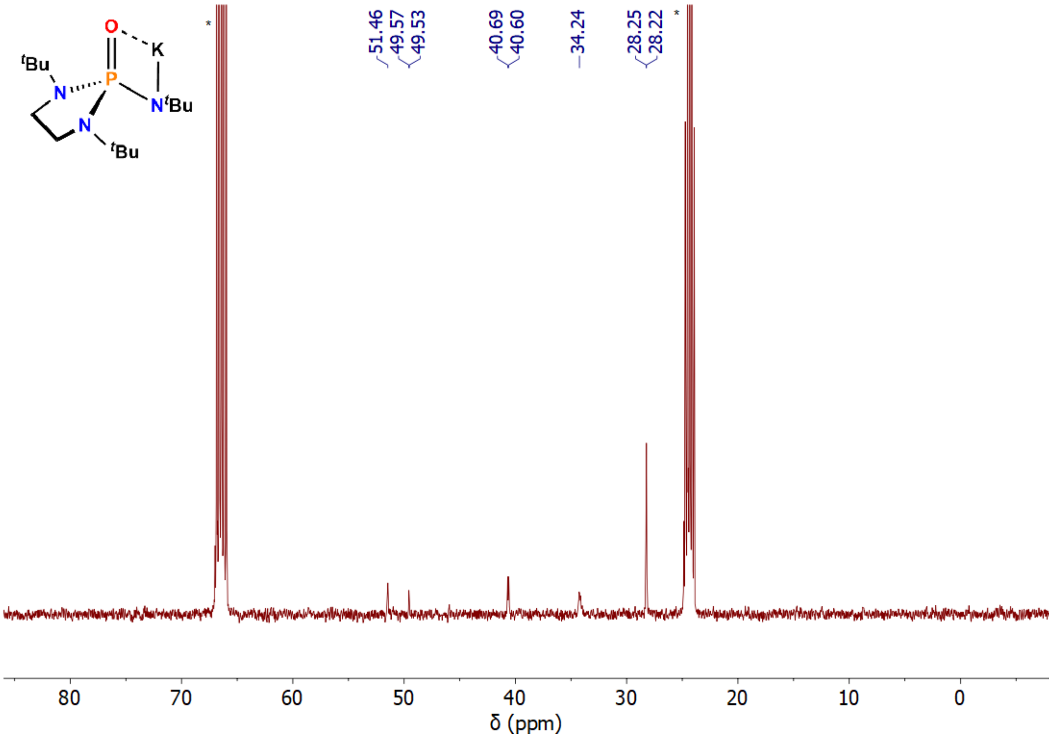

Figure S13. $^{13}C\{^1H\}$ NMR spectrum of **1-K** in THF-d_8. * = THF-d_8

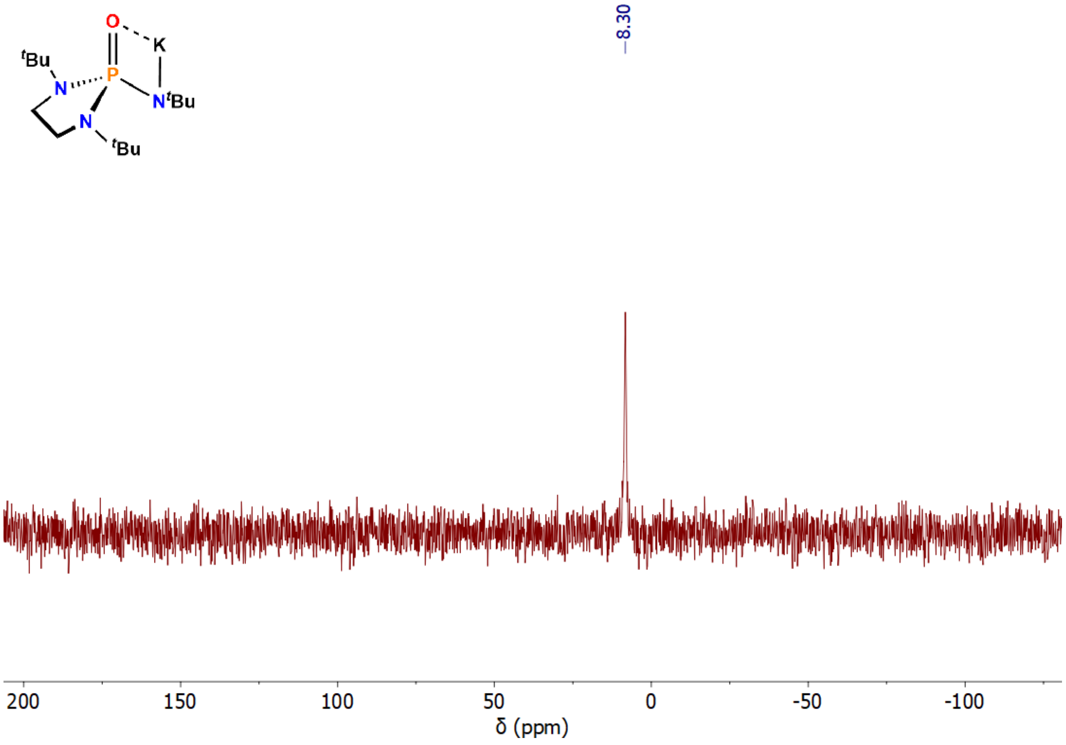

Figure S14. $^{31}P\{^1H\}$ NMR spectrum of **1-K** in THF-d_8.

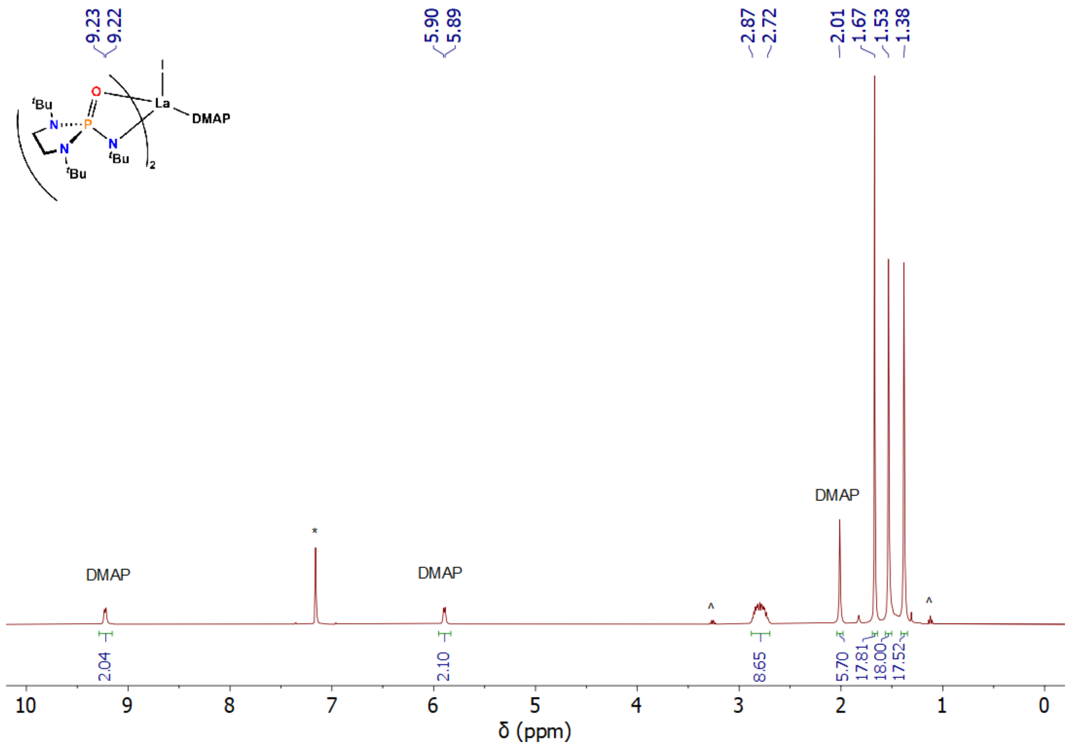

Figure S15. ^{1}H NMR spectrum of **2-LaI** in C_6D_6. * = C_6D_5H, ^ = Et_2O

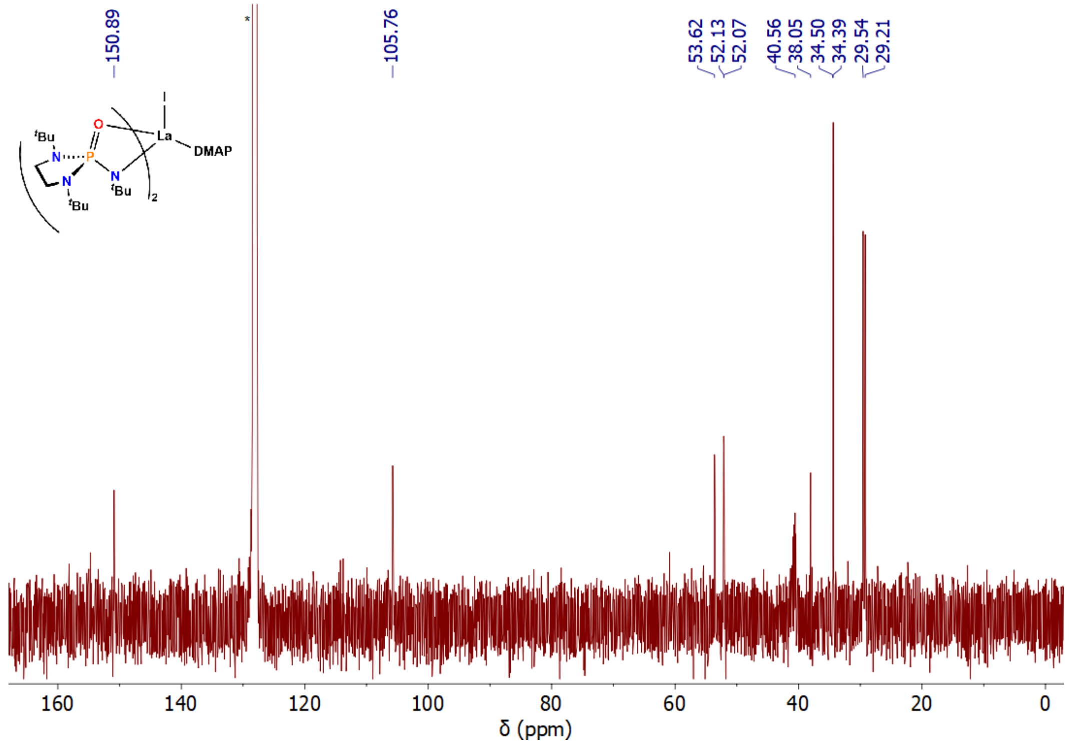

Figure S16. $^{13}C\{^{1}H\}$ NMR of **2-LaI** in C_6D_6. * = C_6D_6

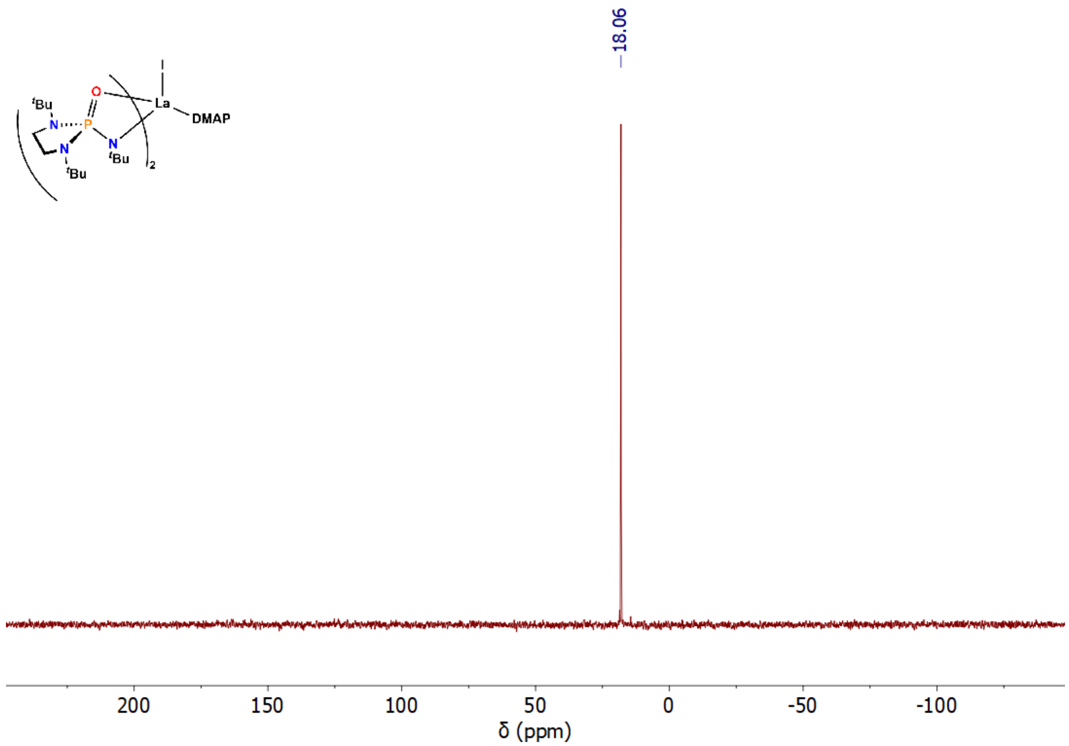

Figure S17. $^{31}P\{^{1}H\}$ NMR spectrum of **2-LaI** in C_6D_6.

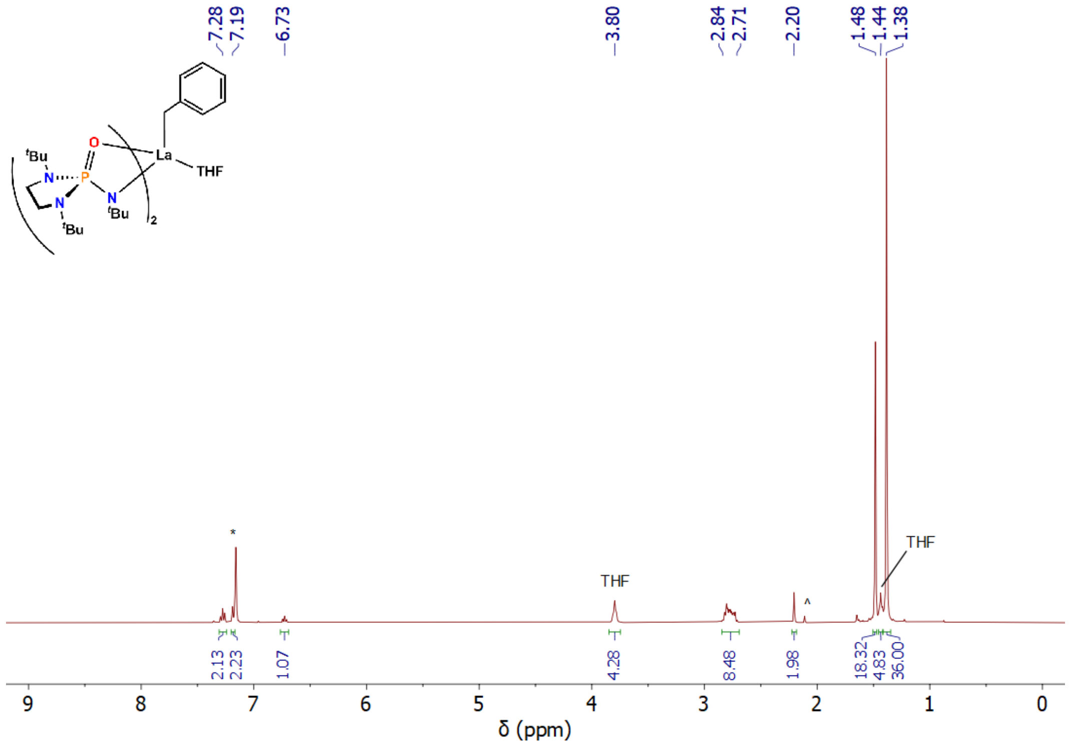

Figure S18. ^{1}H NMR spectrum of **2-LaBn** in C_6D_6. * = C_6D_5H, ^ = toluene

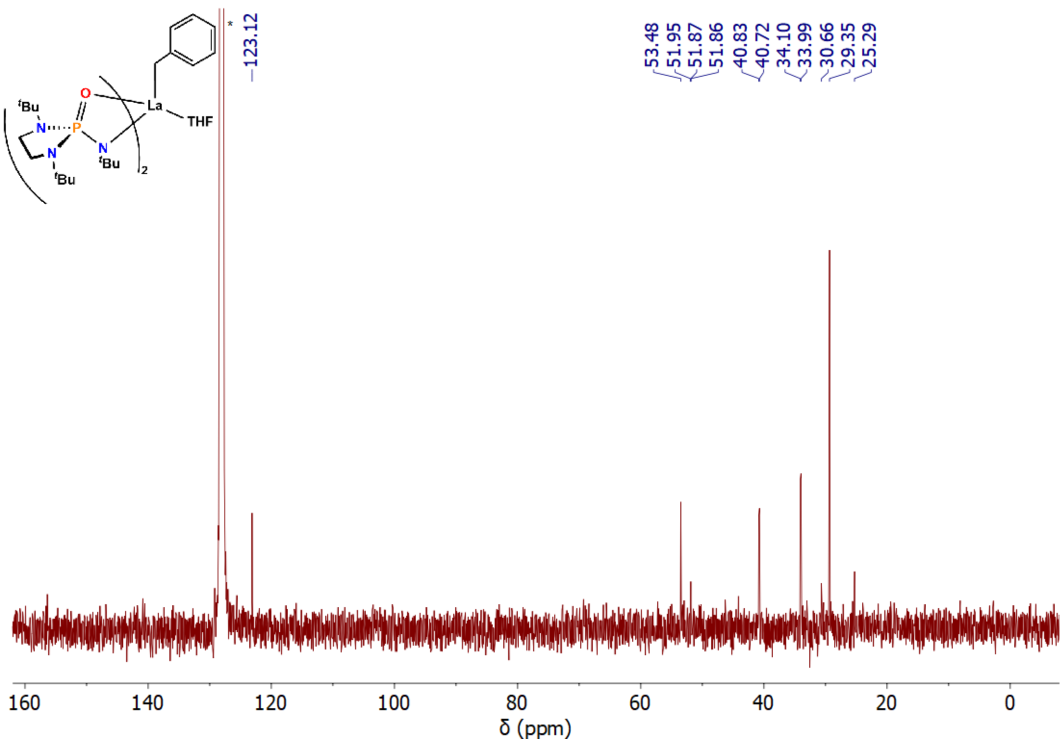

Figure S19. $^{13}C\{^{1}H\}$ NMR spectrum of **2-LaBn** in C_6D_6. * = C_6D_6.

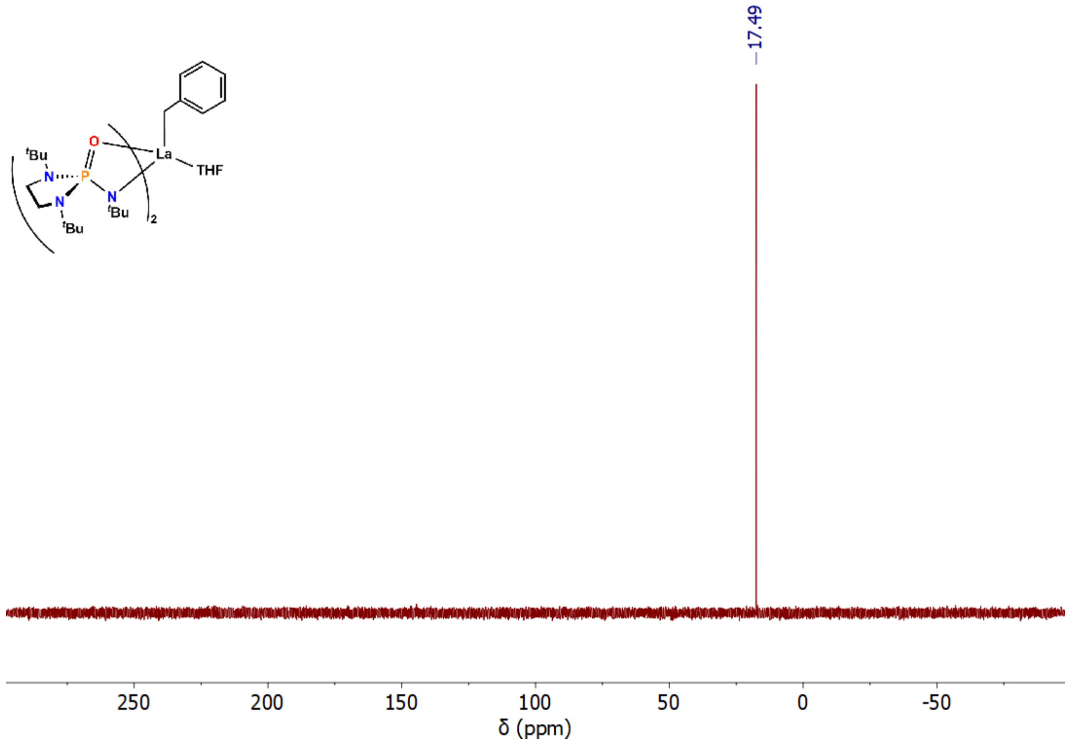

Figure S20. $^{31}P\{^{1}H\}$ NMR spectrum of **2-LaBn** in C_6D_6.

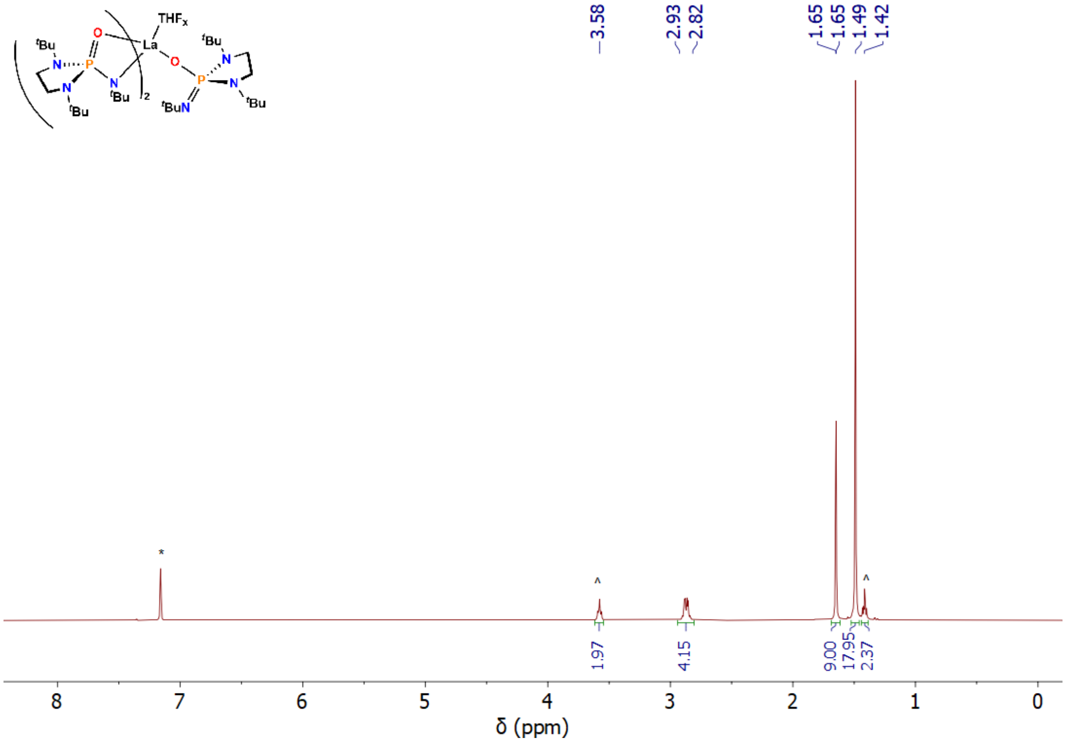

Figure S21. ^{1}H NMR spectrum of **3-La** in C_6D_6. * = C_6D_5H, ^ = THF

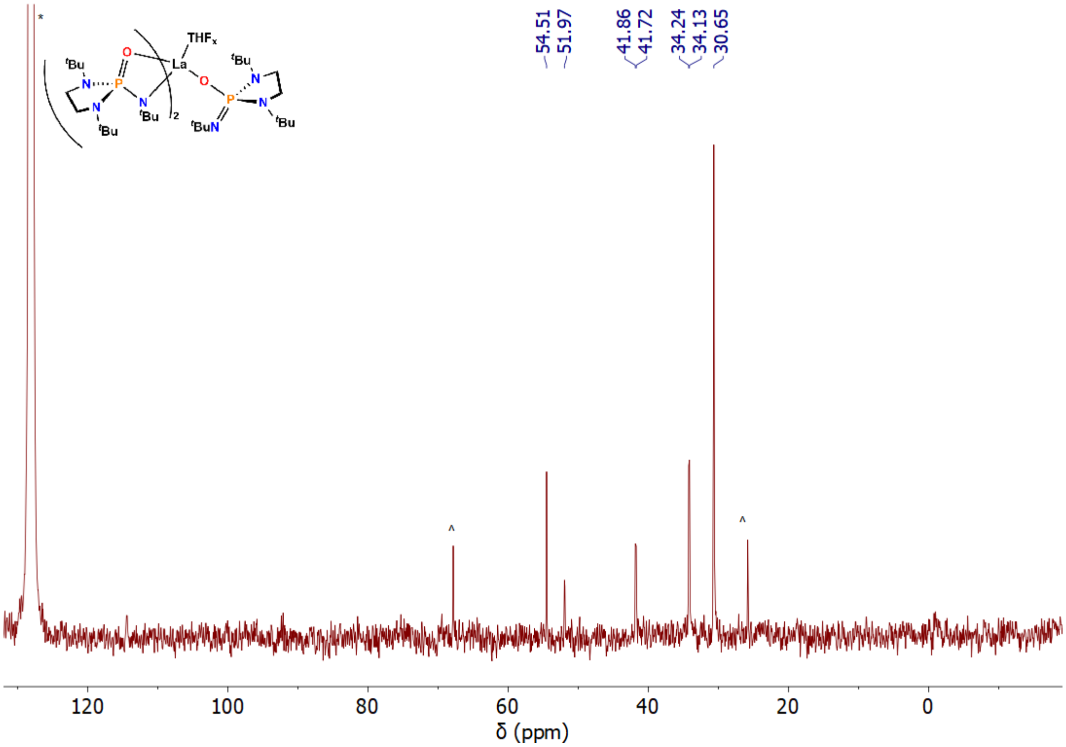

Figure S22. $^{13}C\{^{1}H\}$ NMR spectrum of **3-La** in C_6D_6. * = C_6D_6, ^ = THF

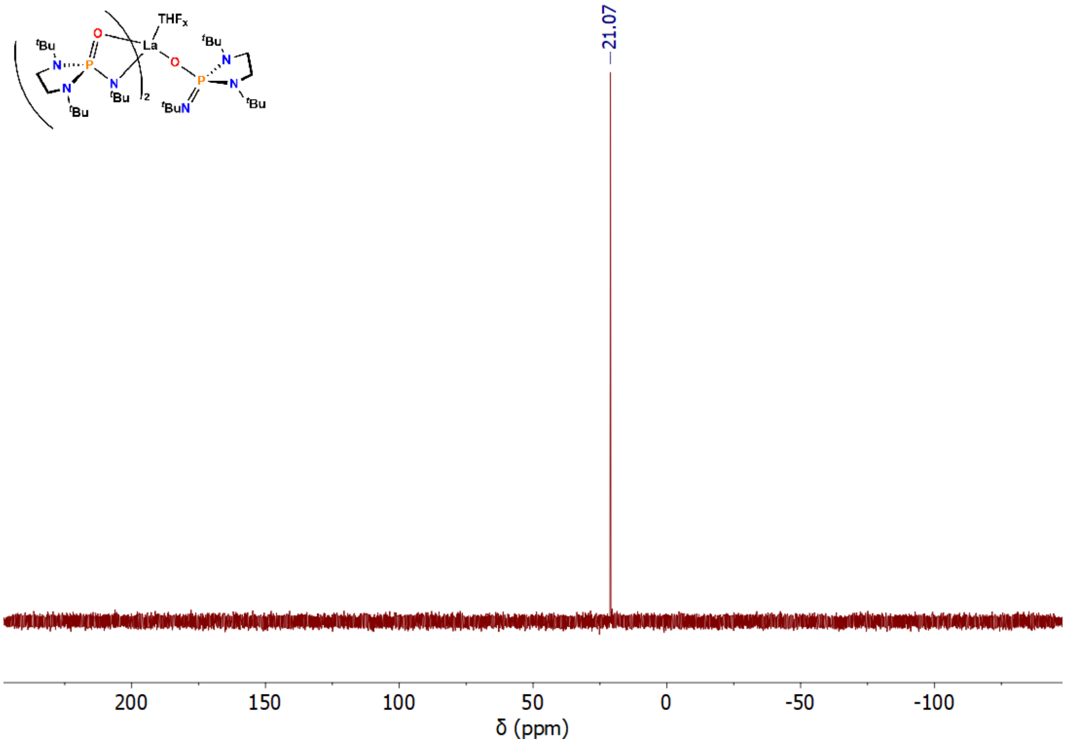

Figure S23. $^{31}P\{^{1}H\}$ NMR spectrum of **3-La** in C_6D_6.

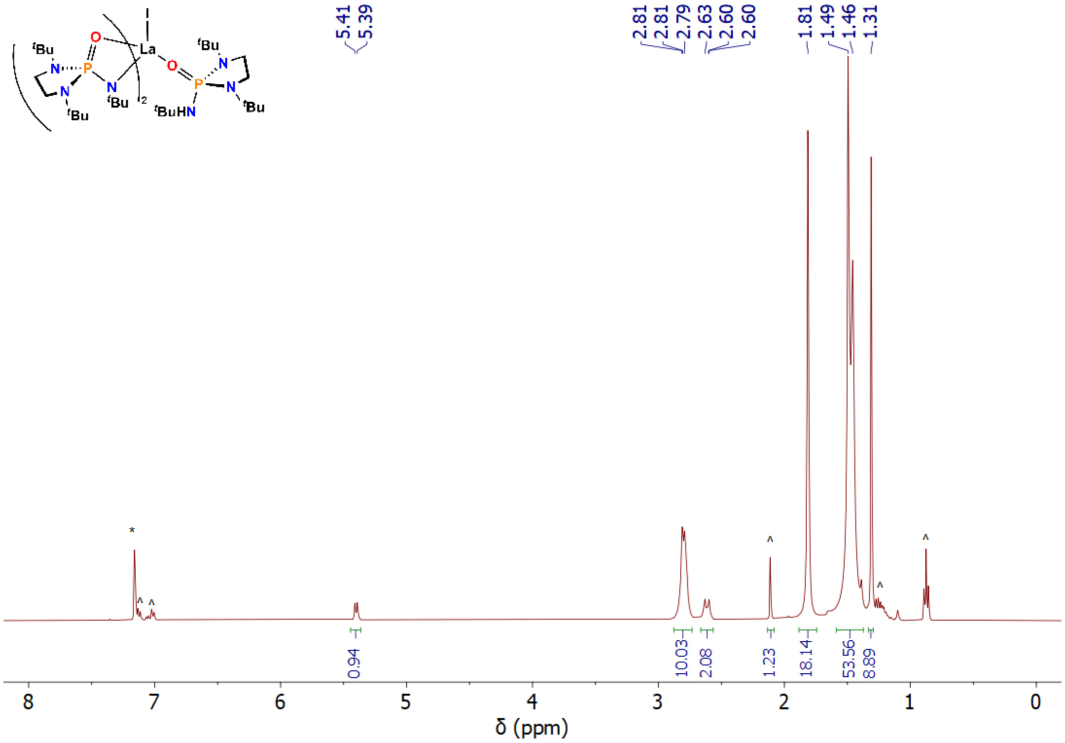

Figure S24. ^{1}H NMR spectrum of **4-LaI** in C_6D_6. * = C_6D_5H, ^ = toluene, *n*-pentane

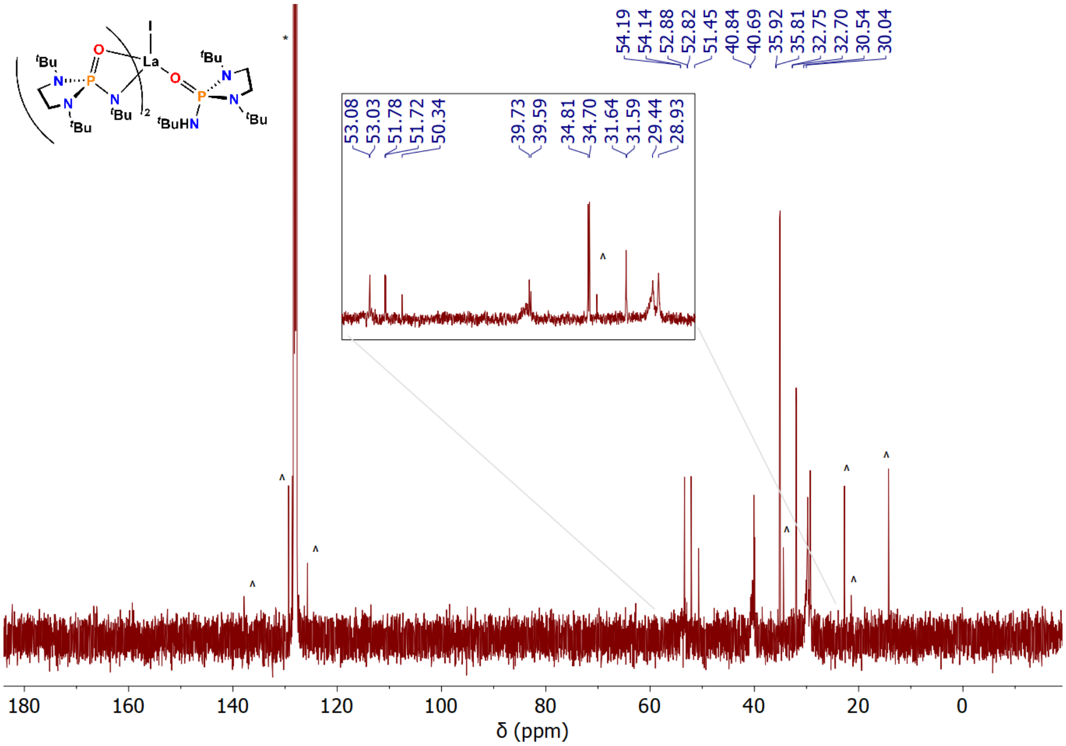

Figure S25. $^{13}C\{^{1}H\}$ NMR spectrum of **4-LaI** in C_6D_6. * = C_6D_6, ^ = toluene, *n*-pentane. Note: Broad feature is present near 40.5 ppm that corresponds to a CH2 (see 13C 135-DEPT) backbone carbon that is too weak to definitively assign a chemical shift. Only 3 out of 4 signals corresponding to quaternary carbons are observed, one is likely broadened similar to resonances at 29.7 and 30.2 ppm.

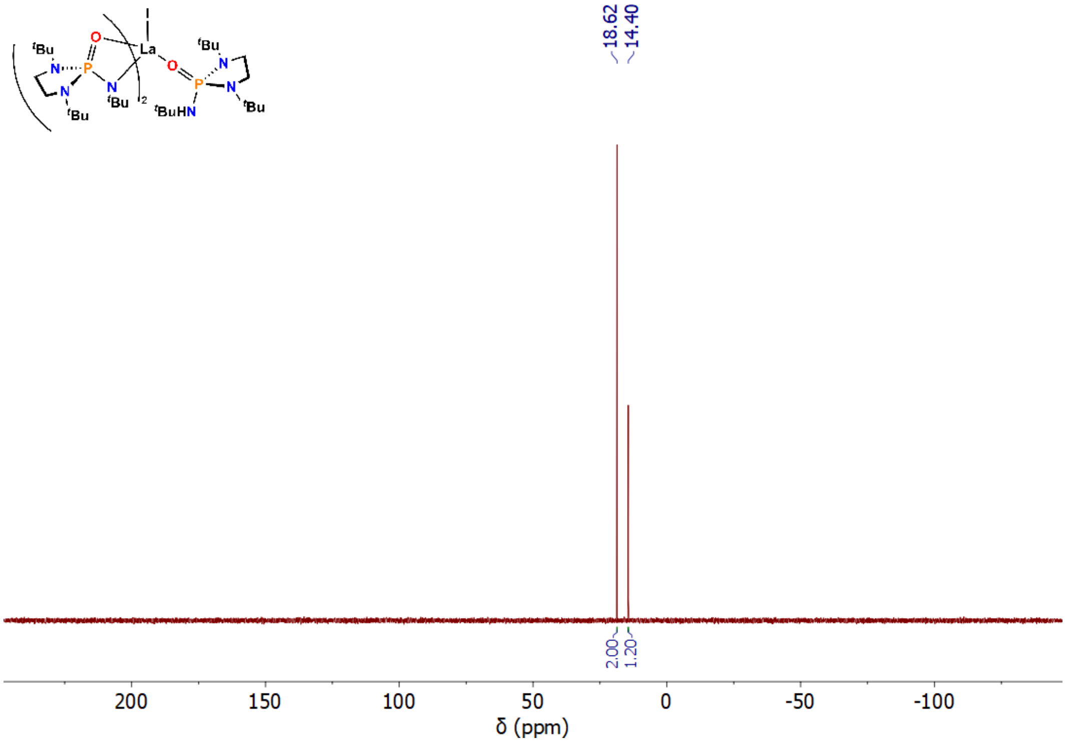

Figure S26. $^{31}P\{^{1}H\}$ NMR spectrum of **4-LaI** in C_6D_6.

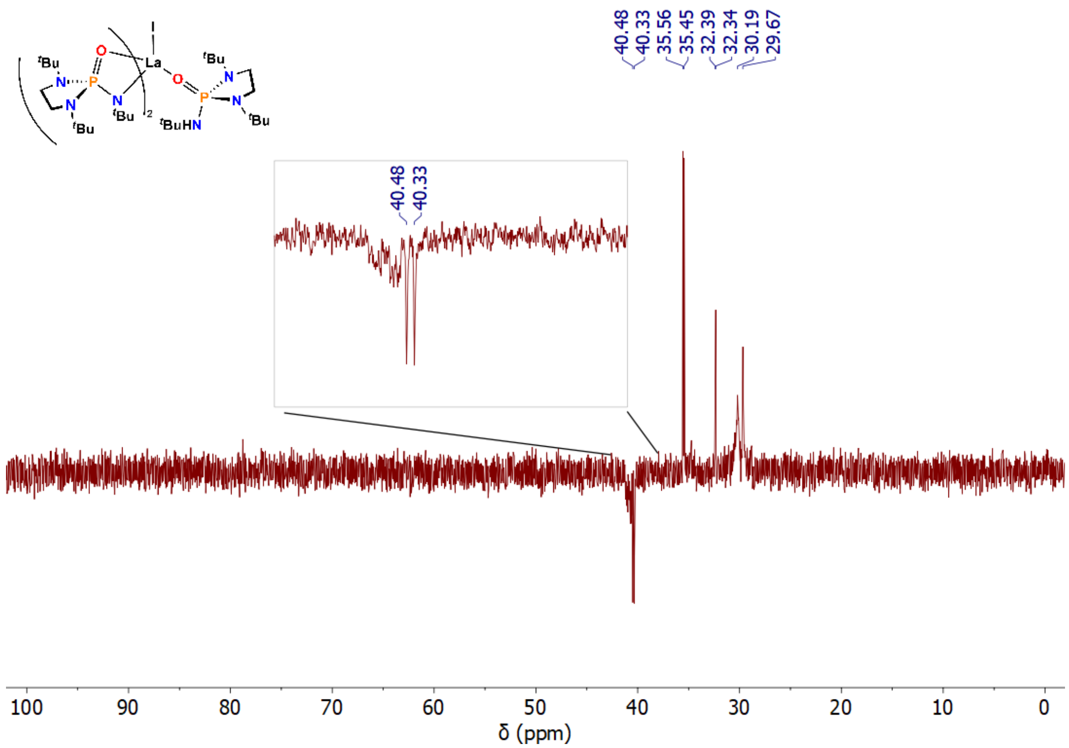

Figure S27. ^{13}C DEPT-135 NMR spectrum of **4-LaI** in C_6D_6.

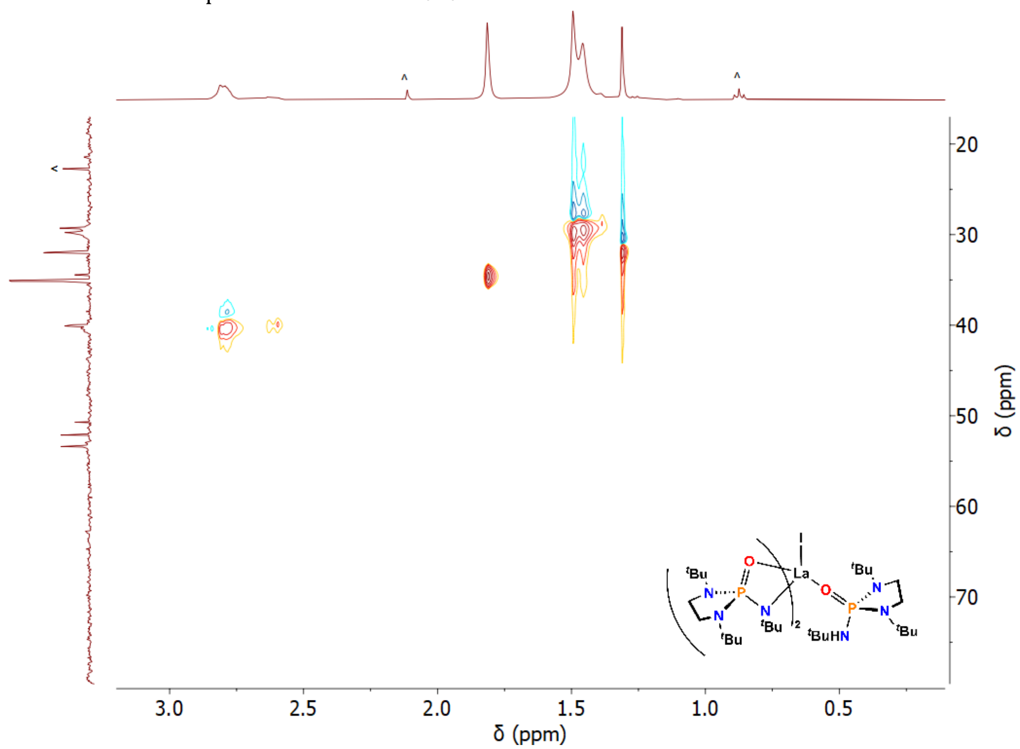

Figure S28. HSQC (^{1}H-^{13}C) NMR spectrum of **4-LaI** in C_6D_6. ^ = toluene, *n*-pentane

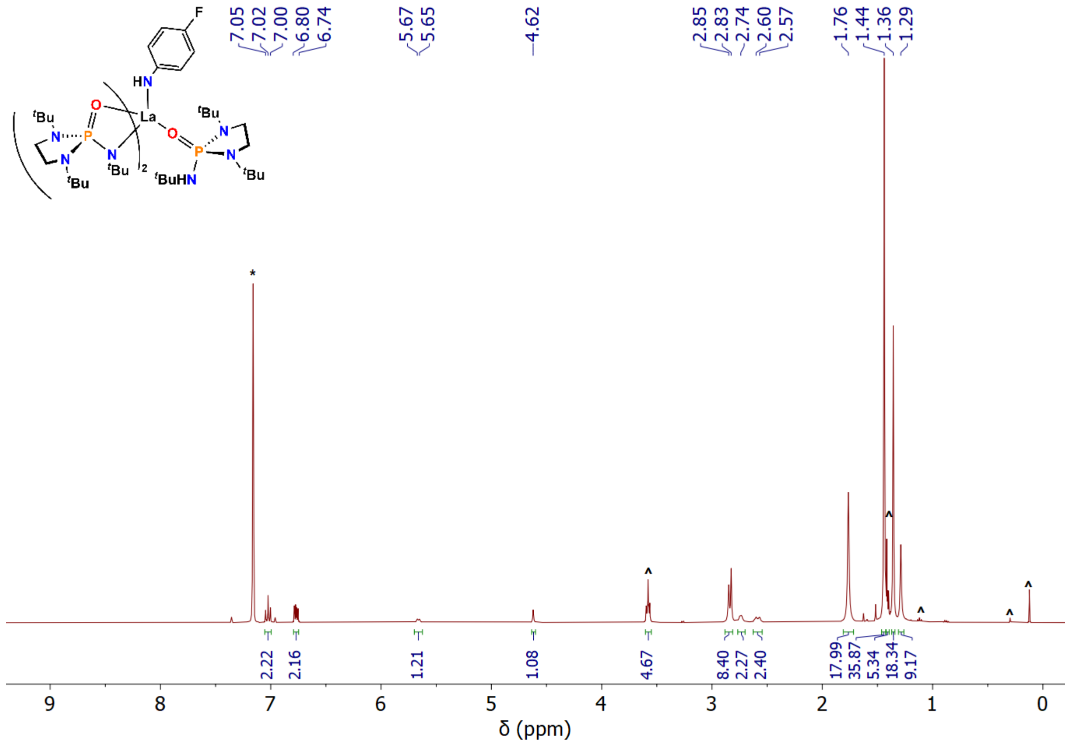

Figure S29. ^{1}H NMR spectrum of **4-La-pF** in C_6D_6. *=C_6D_5H ^ = THF, pentane, silicone grease, HMDSO

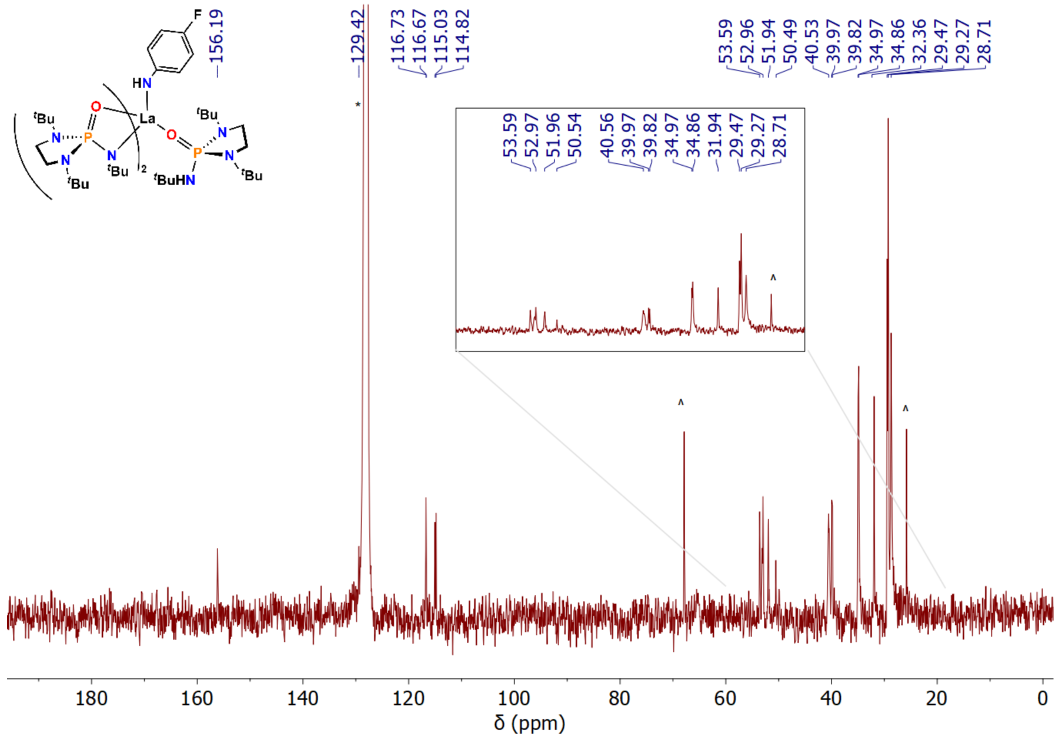

Figure S30. $^{13}C\{^{1}H\}$ NMR spectrum of **4-La-pF** in C_6D_6. * = C_6D_6, ^ = THF

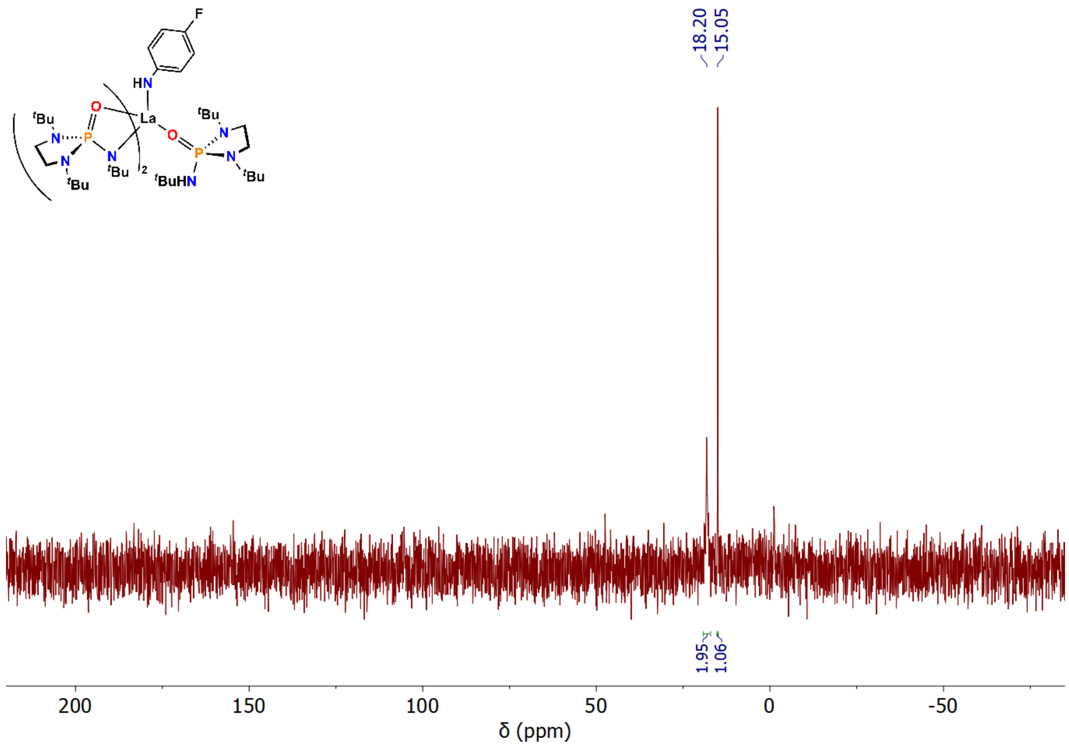

Figure S31. $^{31}P\{^{1}H\}$ NMR spectrum of **4-La-pF** in C_6D_6.

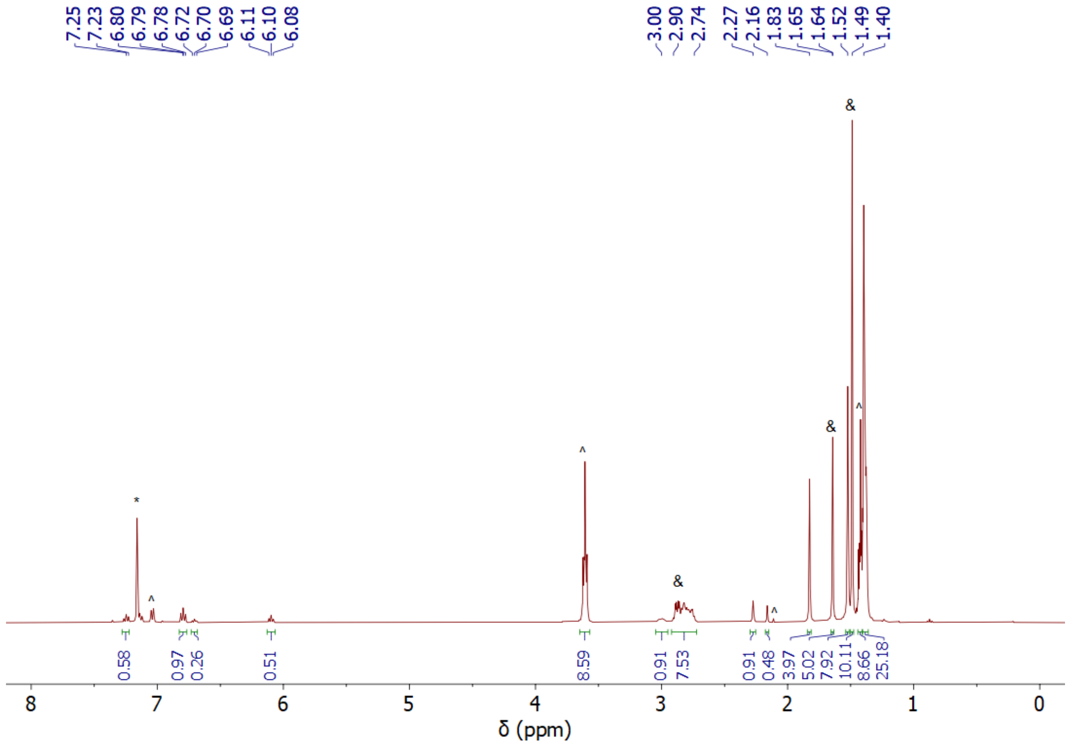

Figure S32. Crude ^{1}H NMR spectrum of reaction between **3-La** and KBn after removal of volatiles in C_6D_6.* = C_6D_5H, ^ = THF, toluene & = **3-La**

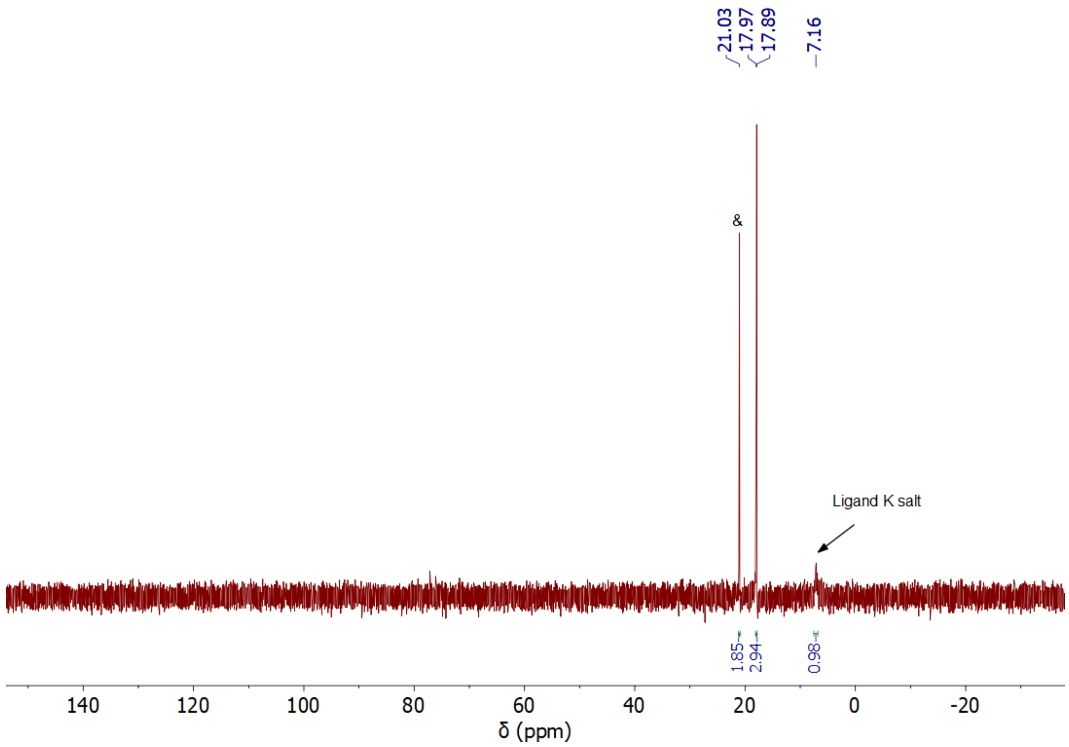

Figure S33. Crude $^{31}P\{^{1}H\}$ NMR spectrum of reaction between **3-La** and KBn after removal of volatiles and dissolution in C_6D_6. & = **3-La**. The signal around 18 ppm is similar to that of **2-LaBn** (17.5 ppm in C_6D_6).

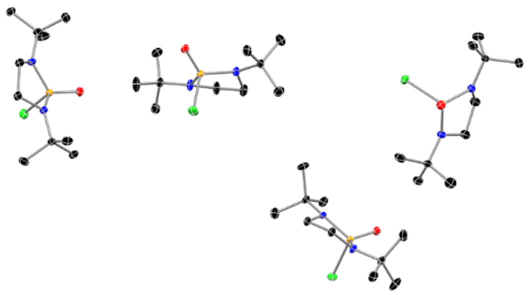

Figure S34. Crystallographic asymmetric unit of **1-Cl**. Thermal ellipsoids drawn at 50% probability level. Hydrogen atoms omitted for clarity.Light green: Cl, orange: P, red: O, blue: N, black: C.

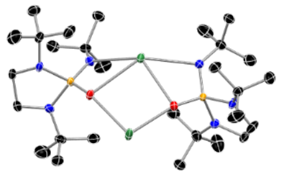

Figure S35. Crystallographic asymmetric unit of **1-K**. Thermal ellipsoids drawn at 50% probability level. Hydrogen atoms omitted for clarity. Green: K, orange: P, red: O, blue: N, black: C.

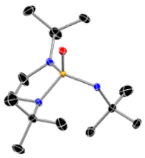

Figure S36. Crystallographic asymmetric unit of **1-H**. Thermal ellipsoids drawn at 50% probability level. Hydrogen atoms omitted for clarity. Orange: P, red: O, blue: N, black, C.

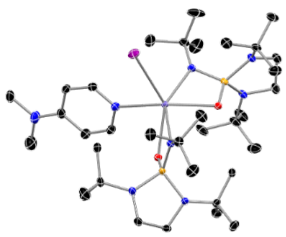

Figure S37. Crystallographic asymmetric unit of **2-LaI**. Thermal ellipsoids drawn at 50% probability level. Hydrogen atoms omitted for clarity. Liliac: La, purple: I, orange: P, red: O, blue: N, black: C.

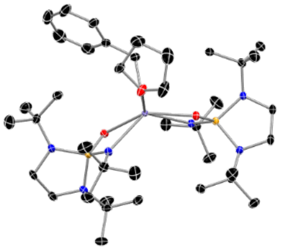

Figure S38. Crystallographic asymmetric unit of **2-LaBn**. Thermal ellipsoids drawn at 50% probability level. Hydrogen atoms omitted for clarity. Liliac: La, orange: P, red: O, blue: N, black: C.

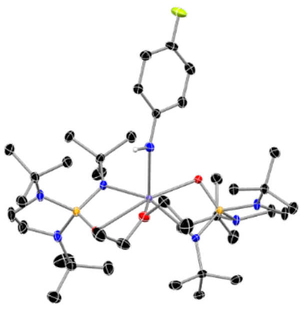

Figure S39. Crystallographic asymmetric unit of **2-La-pF**. Thermal ellipsoids drawn at 50% probability level. Carbon-bound hydrogen atoms omitted for clarity. Liliac: La, orange: P, yellow: F, red: O, blue: N, black: C, white: H.

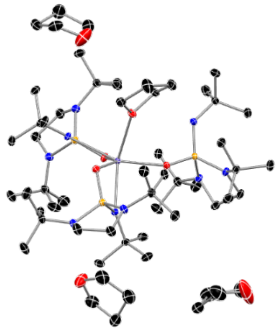

Figure S40. Crystallographic asymmetric unit of **3-La**. Thermal ellipsoids drawn at 50% probability level. Hydrogen atoms omitted for clarity. Liliac: La, orange: P, red: O, blue: N, black: C.

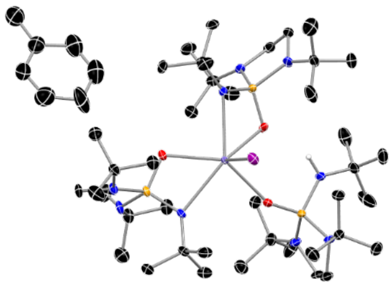

Figure S41. Crystallographic asymmetric unit of **4-LaI**. Thermal ellipsoids drawn at 50% probability level. Carbon-bound hydrogen atoms omitted for clarity. Liliac: La, purple: I, orange: P, yellow: F, red: O, blue: N, black: C, white: H.

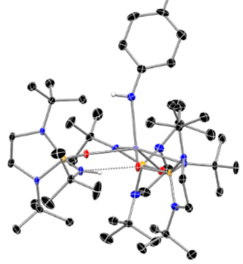

Figure S42. Crystallographic asymmetric unit of **4-La-pF**. Thermal ellipsoids drawn at 50% probability level. Carbon-bound hydrogen atoms omitted for clarity. Liliac: La, orange: P, yellow: F, red: O, blue: N, black: C, white: H.

X-Ray Crystallography

Table S1. Tabulated crystallographic information

	**1-Cl**	**1-H**	**1-K**	**2-LaI**
Formula	$C_{10}H_{22}ClN_2OP$	$C_{14}H_{32}N_3OP$	$C_{14}H_{31}KN_3OP$	$C_{35}H_{72}ILaN_8O_2P_2$
Formula Weight/gmol^{-1}	252.71	289.39	327.49	964.75
Collection Temperature/K	100	100	100	100
Space Group	P2/c	P-1	P-1	$P2_1/c$
System	monoclinic	triclinic	triclinic	monoclinic
Resolution/Å	0.80	0.72	0.72	0.75
a/Å	32.6916(17)	9.3259(6)	13.2143(18)	10.5638(4)
b/Å	6.4911(3)	9.7671(7)	13.2958(18)	22.9095(9)
c/Å	28.7259(13)	11.6550(8)	13.3805(19)	18.6930(7)
α/°	90	94.914(3)	105.424(5)	90
β/°	115.684(2)	112.781(2)	109.874(5)	99.607(2)
γ/°	90	113.854(2)	91.522(5)	90
Volume/Å^3	5493.5(5)	857.45(10)	2113.4(5)	4460.5(3)
Z [Z’]	16[4]	2 [1]	4[2]	4[1]
ρ/gcm^{-3}	1.222	1.121	1.029	1.437
μ/mm^{-1}	0.375	0.159	0.328	1.759
F(000)	2176.0	320.0	712.0	1976.0
Crystal Size/mm^3	0.34x0.239x 0.094	0.327x0.32x 0.272	0.254x0.154x 0.142	0.18x0.144x 0.106
Radiation Type	MoKα (λ = 0.71073)	MoKα (λ = 0.71073)	MoKα (λ = 0.71073)	MoKα (λ = 0.71073)
Physical Description	Colorless plate	Colorless prism	Colorless cube	Colorless plate
Collection 2θ range/°	3.948 to 52.742	3.958 to 59.15	4.296 to 59.148	3.91 to 56.564
Index Ranges	-40≤h≤40, -8≤k≤8, -35≤l≤35	-12≤h≤11, -13≤k≤13, -15≤l≤16	-18≤h≤18, -18≤k≤16, -18≤l≤18	-14≤h≤14, -30≤k≤30, -24≤l≤24
Reflections Collected	250451	16441	51688	190841
Independent Reflections	11209 [R_{int}=0.1355, R_{sigma}=0.0380]	4809 [R_{int}=0.0379, R_{sigma}=0.0359]	11856 [R_{int}=0.0339, R_{sigma}=0.0266]	11076 [R_{int}=0.0806, R_{sigma}=0.0267]
Data/Restraints/Parameters	11209/0/565	4809/0/185	11856/2/383	11076/10/500
Goodness of Fit	1.098	1.070	1.061	1.039
Final R Indices (I ≥2σ)	R1 = 0.0391, wR2 = 0.0855	R1=0.0469, wR2=0.1225	R1 = 0.0459, wR2 = 0.1201	R1 = 0.0220, wR2 = 0.0486
Final R Indices (all data)	R1 = 0.0518, wR2 = 0.0906	R1 = 0.0505, wR2 = 0.1259	R1 = 0.0500, wR2 = 0.1236	R1 = 0.0283, wR2 = 0.0515
Largest Diff. Peak/Hole/eA^3	0.27/-0.40	0.78/-0.53	0.65/-0.45	0.50/-0.73
Completeness to 2θ/%	99.9	99.7	99.7	99.9
Flack Parameter	-	-	-	-
CCDC Number	2299279	2299284	2299285	2299278

Table S2. Tabulated crystallographic information (cont'd)

	**2-LaBn**	**2-La-pF**	**3-La**	**4-LaI**
Formula	$C_{39}H_{77}LaN_6O_3P_2$	$C_{38}H_{77}FLaN_7O_3P_2$	$C_{58}H_{125}LaN_9O_7P_3$	$C_{96}H_{209}I_2La_2N_{18}O_6P_6$
Formula Weight/gmol^{-1}	878.91	899.91	1292.48	2429.24
Collection Temperature/K	100	100	100	100
Space Group	$P2_1/n$	$P2_1/n$	P-1	$P2_12_12_1$
System	monoclinic	monoclinic	triclinic	orthorhombic
Resolution/Å	0.75	0.75	0.70	0.70
a/Å	14.7651(6)	10.5058(3)	13.6518(9)	12.7239(4)
b/Å	19.5909(7)	15.4268(5)	13.6674(8)	20.3032(6)
c/Å	15.9613(6)	27.5034(8)	21.8611(16)	22.9169(7)
α/°	90	90	74.599(2)	90
β/°	105.303(2)	90.3730(10)	79.124(3)	90
γ/°	90	90	60.174(2)	90
Volume/Å^3	4453.3(3)	4457.4	3403.8(4)	5920.3(3)
Z [Z']	4[1]	4[1]	2[1]	2[0.5]
ρ/gcm^{-3}	1.311	1.341	1.261	1.363
μ/mm^{-1}	1.072	1.076	0.751	1.368
F(000)	1856.0	1896.0	1388.0	2538.0
Crystal Size/mm^3	0.342x0.334x 0.21	0.298x0.276x 0.09	0.07x0.07x0.07	0.261x0.204x 0.154
Radiation Type	MoKα (λ = 0.71073)	MoKα (λ = 0.71073)	MoKα (λ = 0.71073)	MoKα (λ = 0.71073)
Physical Description	Colorless block	Colorless plate	Colorless block	Colorless block
Collection 2θ range/°	3.344 to 56.564	3.968 to 56.562	4.49 to 61.03	4.012 to 61.014
Index Ranges	-19≤h≤18, -26≤k≤26, -21≤l≤21	-14≤h≤14, -20≤k≤20, -36≤l≤36	-19≤h≤19, -19≤k≤19, -31≤l≤31	-18≤h≤17, -29≤k≤28, -29≤l≤32
Reflections Collected	163580	157853	126159	94655
Independent Reflections	11048 [R_{int}=0.0820, R_{sigma}=0.0284]	11040 [R_{int}=0.0910, R_{sigma}=0.0318]	20697 [R_{int}=0.0625, R_{sigma}=0.0364]	17976 [R_{int}=0.0918, R_{sigma}=0.0596]
Data/Restraints/Parameters	11048/8/528	11040/0/492	20697/24/787	17976/102/670
Goodness of Fit	1.049	1.076	1.113	1.024
Final R Indices (I ≥2σ)	R1 = 0.0234, wR2 = 0.0596	R1 = 0.0260, wR2 = 0.0592	R1 = 0.0309, wR2 = 0.0760	R1 = 0.0377, wR2 = 0.0849
Final R Indices (all data)	R1 = 0.0268, wR2 = 0.0617	R1 = 0.0313, wR2 = 0.0613	R1 = 0.0377, wR2 = 0.0815	R1 = 0.0471, wR2 = 0.0914
Largest Diff. Peak/Hole/eA^3	0.60/-0.52	0.67/-0.45	1.49/-1.33	1.33/-0.59
Completeness to 2θ/%	100	99.9	99.8	99.9
Flack Parameter	-	-	-	0.006(7)
CCDC Number	2299282	2299277	2299281	2299276

Table S3. Tabulated crystallographic information (cont'd)

	**4-La-pF**
Formula	$C_{52}H_{107}FLaN_{10}O_4P_3$
Formula Weight/gmol^{-1}	1187.29
Collection Temperature/K	100
Space Group	$P2_1/n$
System	monoclinic
Resolution/Å	0.75
a/Å	21.5622(9)
b/Å	13.2868(5)
c/Å	22.4413(9)
α/°	90
β/°	105.657(2)
γ/°	90
Volume/Å^3	6190.7(4)
Z [Z']	4[1]
ρ/gcm^{-3}	1.274
μ/mm^{-1}	0.819
F(000)	2528.0
Crystal Size/mm^3	0.391x0.236x 0.198
Radiation Type	MoKα (λ = 0.71073)
Physical Description	Colorless block
Collection 2θ range/°	3.598 to 56.57
Index Ranges	-28≤h≤28, -17≤k≤17, -29≤l≤29
Reflections Collected	248012
Independent Reflections	15374 [R_{int}=0.0970, R_{sigma}=0.0321]
Data/Restraints/Parameters	15374/18/705
Goodness of Fit	1.059
Final R Indices (I ≥2σ)	R1 = 0.0295, wR2 = 0.0704
Final R Indices (all data)	R1 = 0.0360, wR2 = 0.0735
Largest Diff. Peak/Hole/eA^3	0.91/-1.02
Completeness to 2θ/%	100
Flack Parameter	-
CCDC Number	2299280

Refinement Details

Crystals suitable for X-ray diffraction were coated in paratone or Cargille-NVH oil in a glovebox and transferred to the diffractometer in a 20 mL capped vial. Crystals were mounted on a nylon loop on a Bruker D8 VENTURE diffractometer dual wavelength Mo/Cu four-circle diffractometer with a microfocus sealed X-ray tube using a mirror optics as monochromator and a Bruker PHOTON II detector. The diffractometer is equipped with an Oxford Cryostream 800 cryostat and crystals were cooled and kept at T = 100(2) K during data collection. All data were integrated with SAINT and a multi-scan absorption correction using SADABS was applied.[1] The structures were solved with the ShelXT structure solution program using the Intrinsic Phasing solution method and refined by full-matrix least-squares methods against F^2 using SHELXL-2014[2,3] and by using Olex2 1.5-alpha[4] as the graphical interface. All non-hydrogen atoms were refined with anisotropic displacement parameters. All hydrogen atoms were refined isotropically on calculated positions using a riding model with their Uiso values constrained to 1.5 times the Ueq of their pivot atoms for terminal sp3 carbon atoms and 1.2 times for all other carbon atoms. Disordered moieties were refined using bond lengths restraints and displacement parameter restraints. Crystallographic data for the structures reported in this paper have been deposited with the Cambridge Crystallographic Data Centre.

Table S4:.Select bond distances for **1-Cl**

Atoms	Distance	ESD	Atoms	Distance	ESD
P1-O1	1.4638	0.0016	P1-N1	1.6305	0.0019
P2-O2	1.4667	0.0015	P1-N2	1.633	0.002
P3-O3	1.4626	0.0014	P2-N3	1.641	0.002
P4-O4	1.4654	0.0017	P2-N4	1.634	0.002
P1-Cl1	2.0906	0.001	P3-N5	1.631	0.002
P2-Cl2	2.0758	0.0008	P3-N6	1.628	0.002
P3-Cl3	2.0915	0.0007	P4-N7	1.6365	0.0019
P4-Cl4	2.0738	0.001	P4-N8	1.635	0.0018

Table S5. Select bond distances for **1-H**

Atoms	Distance	ESD
P-O	1.490	0.001
P1-N3	1.638	0.002
N3-C11	1.477	0.002
N1-P1	1.675	0.001
N2-P1	1.672	0.001

Table S6. Select bond distances for **1-K**

Atoms	Distance	ESD
P1-O1	1.522	0.001
P1-N3	1.561	0.002
P1-N2	1.736	0.001
P1-N1	1.707	0.001
P2-O2	1.518	0.001
P2-N6	1.574	0.001
P2-N4	1.693	0.002
P2-N5	1.706	0.001

Table S7. Select bond distances for **2-LaI**

Atoms	Distance	ESD	Atoms	Distance	ESD
La1-O1	2.4197	0.0012	P1-N1	1.5919	0.0016
La1-O2	2.3847	0.0013	P2-N4	1.5973	0.0016
La-N1	2.541	0.0015	P1-N2	1.6759	0.0015
La-N4	2.5536	0.0016	P1-N3	1.6788	0.0018
La1-I1	3.2057	0.0005	P2-N5	1.6668	0.0017
La1-N7	2.6861	0.0018	P2-N6	1.6757	0.017
P1-O1	1.5405	0.0014	O1-P1-N1	102.27	0.07
P2-O2	1.5348	0.0012	O2-P2-N4	102.27	0.08

Table S8. Select bond distances for **2-LaBn**

Atoms	Distance	ESD	Atoms	Distance	ESD
La1-O1	2.4915	0.0012	P2-O2	1.5309	0.0011
La1-O2	2.4579	0.001	P1-N1	1.6047	0.0012
La-N1	2.5217	0.0014	P2-N4	1.6062	0.0012
La-N4	2.5265	0.0013	P1-N2	1.6919	0.0016
La1-C29	2.65	0.002	P1-N3	1.6742	0.0014
La1-C30	3.715	0.002	P2-N5	1.6729	0.0016
La1-O3	2.5846	0.0012	P2-N6	1.6715	0.0017
P1-O1	1.5283	0.0012			

Table S9. Select bond distances for **2-La-pF**

Atoms	Distance	ESD	Atoms	Distance	ESD
La1-O1	2.5051	0.0012	P2-N4	1.6061	0.0015
La1-O2	2.4557	0.0012	P1-N2	1.6737	0.0017
La-N1	2.4991	0.0015	P1-N3	1.6844	0.0015
La-N4	2.5351	0.0015	P2-N5	1.6666	0.0015
La1-O3	2.6908	0.0014	P2-N6	1.6935	0.0015
P1-O1	1.528	0.0013	La-N10	2.4134	0.0017
P2-O2	1.528	0.0013	N10-C43	1.379	0.003
P1-N1	1.5985	0.0015			

Table S10. Select bond distances for **3-La**

Atoms	Distance	ESD	Atoms	Distance	ESD
La1-O1	2.5282	0.0016	P1-N2	1.6969	0.0013
La1-O2	2.4194	0.0017	P1-N3	1.67	0.002
La-N1	2.5198	0.0012	P2-N5	1.6754	0.0018
La-N4	2.6082	0.0014	P2-N6	1.6854	0.0014
La1-O3	2.3479	0.0012	P3-O3	1.5485	0.0013
P1-O1	1.5254	0.001	P3-N7	1.5599	0.0011
P2-O2	1.5274	0.0011	P3-N8	1.71	0.002
P1-N1	1.6073	0.0019	P3-N9	1.7041	0.0016
P2-N4	1.604	0.0019			

Table S11. Select bond distances for **4-LaI**

Atoms	Distance	ESD	Atoms	Distance	ESD
La1-O1	2.404	0.003	P2-N4	1.592	0.004
La1-O2	2.455	0.003	P1-N3	1.667	0.004
La-N1	2.6	0.004	P1-N2	1.69	0.004
La-N4	2.591	0.004	P2-N5	1.68	0.004
La1-O3	2.451	0.004	P2-N6	1.681	0.004
La1-I1	3.1887	0.0006	P3-O3	1.508	0.004
P1-O1	1.535	0.004	P3-N7	1.626	0.005
P2-O2	1.53	0.003	P3-N8	1.648	0.004
P1-N1	1.598	0.004	P3-N9	1.663	0.004

Table S12. Select bond distances for **4-La-pF**

Atoms	Distance	ESD	Atoms	Distance	ESD
La1-O1	2.4784	0.0014	P1-N3	1.6886	0.0015
La1-O2	2.4291	0.0012	P2-N5	1.6706	0.0015
La-N1	2.6132	0.0018	P2-N6	1.6834	0.0016
La-N4	2.6034	0.0016	P3-O3	1.5078	0.0013
La1-O3	2.4802	0.0014	P3-N7	1.6311	0.0018
P1-O1	1.5333	0.0016	P3-N8	1.6638	0.0017
P2-O2	1.5284	0.0015	P3-N9	1.6642	0.0015
P1-N1	1.5872	0.0015	La-N10	2.3959	0.0016
P2-N4	1.5974	0.0016	N10-C43	1.377	0.002
P1-N2	1.671	0.0017			

Table S13. Select bond angles for **1-Cl**

1	C7	N2	P1	128.2(2)
2	P1	N2	C6	108.7(1)
3	C6	N2	C7	118.7(2)
4	C5	N1	C1	119.3(2)
5	C1	N1	P1	125.9(2)
6	P1	N1	C5	111.2(2)
7	C11	N3	P2	126.9(2)
8	P2	N3	C15	108.6(1)
9	C15	N3	C11	118.8(2)
10	P2	N4	C17	124.2(2)
11	C17	N4	C16	119.1(2)
12	C16	N4	P2	111.1(1)
13	P3	N5	C21	128.1(2)
14	C21	N5	C25	118.8(2)
15	C25	N5	P3	109.0(2)
16	P3	N6	C27	126.5(2)
17	C27	N6	C26	119.0(2)
18	C26	N6	P3	111.0(2)
19	P4	N8	C37	124.4(2)
20	C37	N8	C36	119.0(2)
21	C36	N8	P4	111.1(1)
22	P4	N7	C35	108.7(1)
23	C35	N7	C31	118.7(2)
24	C31	N7	P4	127.8(2)

Table S14.-Select bond angles for **1-H**

Atom1	Atom2	Atom3	Angle
C1	N1	C5	118.8(1)
C5	N1	P1	111.9(1)
P1	N1	C1	124.4(1)
P1	N2	C6	110.5(1)
C6	N2	C7	116.2(1)
C7	N2	P1	123.4(1)

Table S15. Select bond angles for **1-K**

Atom1	Atom2	Atom3	Angle
C15	N4	P2	128.0(1)
P2	N4	C19	113.5(1)
C19	N4	C15	117.6(1)
C20B	N5	C21	119.2(3)
C21	N5	C20A	116.2(1)
C20A	N5	P2	109.3(1)
C20B	N5	P2	115.6(3)
P2	N5	C21	124.9(1)
C1	N1	C5	116.0(1)
C5	N1	P1	112.6(1)
P1	N1	C1	123.8(1)
P1	N2	C6	109.6(1)
C6	N2	C7	116.2(1)
C7	N2	P1	123.8(1)

Table S16. Select bond angles for **2-LaBn**

Atom1	Atom2	Atom3	Angle
P1	N2	C11	122.5(1)
C11	N2	C10	115.2(1)
P1	N2	C10	111.1(1)
P1	N3	C5	128.4(1)
C5	N3	C9	118.1(1)
C9	N3	P1	112.3(1)
C25	N6	P2	127.2(1)
P2	N6	C24	112.9(3)
C24	N6	C25	118.8(3)
P2	N5	C19	125.7(1)
C19	N5	C23	114.9(2)
C23	N5	P2	113.3(2)
C24A	N6	C25	115(1)
C24A	N6	P2	114(1)
C23A	N5	C19	123(1)
C23A	N5	P2	110.8(9)

Table S17. Select bond angles for **2-La-pF**

Atom1	Atom2	Atom3	Angle
C25	N6	C24	115.0(1)
C24	N6	P2	110.7(1)
P2	N6	C25	122.7(1)
C23	N5	P2	113.1(1)
P2	N5	C19	128.5(1)
C19	N5	C23	118.2(1)
C11	N3	C10	116.2(2)
C10	N3	P1	111.3(1)
P1	N3	C11	126.2(1)
C9	N2	P1	111.8(1)
P1	N2	C5	129.9(1)
C5	N2	C9	118.3(2)

Table S18. Select bond angles for **3-La**

Atom1	Atom2	Atom3	Angle
C11	N3	P1	128.6(1)
P1	N3	C10	113.0(1)
C10	N3	C11	117.9(2)
P1	N2	C5	123.3(1)
C5	N2	C9	114.8(1)
C9	N2	P1	110.3(1)
C33	N8	P3	128.4(1)
P3	N8	C37	109.7(2)
C37	N8	C33	114.7(2)
P3	N9	C39	128.1(1)
C39	N9	C38	117.9(2)
C38	N9	P3	112.9(2)
C37A	N8	C33	120.1(8)
C37A	N8	P3	111.3(8)
C38A	N9	P3	112.6(9)
C38A	N9	C39	111.0(9)
P2	N6	C25	128.8(1)
C25	N6	C24	116.9(2)
C24	N6	P2	110.5(2)
P2	N5	C23	112.9(2)
C23	N5	C19	118.9(2)
C19	N5	P2	126.4(1)
C24A	N6	C25	115.0(6)
C24A	N6	P2	116.1(6)
C23A	N5	P2	107.9(4)
C23A	N5	C19	112.7(4)

Table S19. Select bond angles for **4-LaI**

Atom1	Atom2	Atom3	Angle
C33	N8	P3	128.7(3)
P3	N8	C37	112.8(3)
C37	N8	C33	118.5(4)
P3	N9	C38	109.8(3)
C38	N9	C39	116.2(4)
P3	N9	C39	124.8(3)
C25	N6	P2	129.6(3)
P2	N6	C24	112.2(3)
C24	N6	C25	117.3(4)
P2	N5	C23	111.1(3)
C23	N5	C19	115.7(4)
C19	N5	P2	127.9(3)
C5	N2	P1	126.6(3)
P1	N2	C9	110.3(3)
C9	N2	C5	116.7(4)
P1	N3	C11	128.8(3)
C11	N3	C10	118.0(4)
C10	N3	P1	113.1(3)

Table S20. Select bond angles for **4-La-pF**

Atom1	Atom2	Atom3	Angle
C11	N3	P1	123.5(1)
P1	N3	C10	109.6(1)
C10	N3	C11	116.6(1)
P1	N2	C5	128.4(1)
C5	N2	C9	118.6(2)
C9	N2	P1	112.9(1)
C25	N6	P2	124.3(1)
P2	N6	C24	109.9(2)
C24	N6	C25	115.1(2)
P2	N5	C19	126.0(1)
C19	N5	C23	119.3(2)
C24A	N6	C25	121.4(6)
C24A	N6	P2	113.1(6)
C23A	N5	C19	115.7(6)
C23A	N5	P2	109.2(6)
P3	N9	C39	126.5(1)
C39	N9	C38	119.1(4)
C38	N9	P3	112.9(4)
C37	N8	P3	109.5(5)
P3	N8	C33	126.5(1)
C33	N8	C37	116.1(5)
C38A	N9	C39	115.3(3)
C38A	N9	P3	108.5(3)
C37A	N8	C33	119.4(4)
C37A	N8	P3	112.9(4)

References

1. Krause, L., Herbst-Irmer, R., Sheldrick, G., Stalke, D., *J. Appl .Crystallogr.* **2015**, 48, 3-10.

2 Bruker, V8.40B ed., Bruker AXS Inc., Madison, Wisconsin, USA.

3. Sheldrick, G. *Acta Crystallogr.. A* **2015**, 71, 3-8.

4. Dolomanov, O., Bourhis, L., Gildea, R., Howard, J., Puschmann, H., *J. Appl. Crystallogr*. **2009**, 42, 339-341.
